# Multi-omics characterization of RNA modification enzymes identifies NAT10 as a functionally validated prognostic biomarker in hepatocellular carcinoma

**DOI:** 10.3389/fimmu.2026.1764106

**Published:** 2026-01-28

**Authors:** Qianqian Zhan, Huihui Sun, Xiangting Wang, Xiaolin Liang

**Affiliations:** 1 Division of Life Sciences and Medicine, School of Life Sciences, University of Science and Technology of China, Hefei, Anhui, China; 2 Department of Geriatrics, Gerontology Institute of Anhui Province, Centre for Leading Medicine and Advanced Technologies of IHM, The First Affiliated Hospital of USTC, Division of Life Sciences and Medicine, University of Science and Technology of China, Hefei, Anhui, China; 3 Anhui Provincial Key Laboratory of Tumor Immunotherapy and Nutrition Therapy, Hefei, Anhui, China

**Keywords:** copy number variation, hepatocellular carcinoma, immune microenvironment, NAT10, prognostic signature, RNA modification enzymes, single-cell transcriptomics, tumor immunotherapy

## Abstract

**Background:**

RNA modification enzymes (RMEs) are key post-transcriptional regulators that impact RNA stability, translation, and splicing. Dysregulation of RMEs is closely associated with tumor initiation and progression. However, their global regulatory patterns and clinical relevance across cancer types remain incompletely characterized.

**Methods:**

We conducted an integrative multi-omics analysis of RME expression, copy number variation (CNV), and clinical outcomes across multiple cancers. Machine learning algorithms were employed to identify tumor-discriminating RME signatures. Single-cell RNA sequencing (scRNA-seq) characterized tumor microenvironmental heterogeneity. A LASSO-derived prognostic model was established and validated in independent cohorts. Drug sensitivity prediction and supportive functional assays (EdU assays, qRT-PCR, immunohistochemistry) were performed for representative RMEs.

**Results:**

RMEs were broadly upregulated across cancers and showed strong associations with CNV gains. Machine learning identified 12 RMEs that reliably discriminated tumor from normal tissues. Single-cell transcriptomic analysis showed that 10 of the 12 selected RMEs (DKC1, METTL1, NAT10, TRMT1, RPUSD1, PUS1, WDR4, TRMU, ADAT2, GTPBP3) exhibited higher expression in tumor-infiltrating cells compared with adjacent normal tissues. T-cell subpopulations displayed marked heterogeneity, with ADAT2 preferentially enriched in regulatory T cells. CellChat analysis revealed T cell subsets as key mediators of intercellular communication via multiple immune-related pathways. A 6-gene prognostic model exhibited independent prognostic power and was integrated into a well-calibrated nomogram. Drug-response prediction revealed that high-risk patients exhibited enhanced sensitivity to microtubule-targeting agents and kinase inhibitors, whereas low-risk patients showed preferential response to epigenetic modulators. Importantly, supportive functional assays showed that NAT10 knockdown, validated by qRT-PCR, was associated with reduced proliferative activity in HCC cells as evidenced by EdU assays, and IHC validation further corroborated its overexpression in clinical tumor specimens compared to adjacent normal tissues.

**Conclusions:**

This study delineates a CNV-associated landscape of RME dysregulation across cancers and establishes a 12-RME diagnostic signature and a 6-gene prognostic model with robust predictive performance. Single-cell analyses reveal tumor- and cell-type-specific expression patterns of RMEs, while supportive functional data suggest a potential biological relevance of NAT10 in HCC. Collectively, these findings provide an association-based framework for understanding the potential roles of RNA modification programs in cancer progression and clinical stratification.

## 1 Introduction

As one of the leading causes of death worldwide, cancer originates from intricate interactions among genetic, epigenetic, and environmental factors (1). Epigenetic regulation plays a fundamental role in cancer development by shaping gene expression programs without altering the underlying DNA sequence (2). Beyond DNA methylation and histone modifications, RNA modifications have emerged as an essential post-transcriptional regulatory layer that governs RNA stability, translation efficiency, and cellular stress responses (3). A growing body of evidence indicates that aberrant expression of RNA modification enzymes (RMEs) is associated with tumor initiation, metabolic reprogramming, therapeutic resistance, and immune evasion across diverse malignancies (4). Together, these findings highlight RNA modification as an important regulatory layer that contributes to cancer-associated transcriptional and phenotypic heterogeneity.

HCC, accounting for 75-85% of all primary liver cancers, remains one of the most lethal malignancies worldwide (5). Despite considerable advances in surgical and systemic therapies, the prognosis of HCC patients remains poor (6, 7), underscoring the need for improved prognostic models and effective molecular targets. Molecular profiling studies have revealed that HCC exhibits marked genomic instability, including frequent copy-number gains and losses that profoundly reshape transcriptional programs (8, 9). CNV amplification represents a major mechanism of oncogenic activation in HCC, contributing to dysregulated cell-cycle control, metabolic reprogramming, and immune evasion (10). Recent comparative genomic analyses indicate that HCC is characterized by a particularly high burden of copy-number alterations, highlighting its reliance on dosage-sensitive oncogenic programs (11). Given this CNV-enriched genomic landscape, dissecting CNV-associated RMEs could uncover key regulators of HCC progression and identify novel prognostic biomarkers or therapeutic targets.

To systematically evaluate RNA modification enzymes with potential relevance to hepatocellular carcinoma, we performed a comparative analysis of CNV-associated RMEs across multiple cancer types. This analysis revealed that, among all evaluated malignancies, HCC harbors the highest proportion of CNV-associated RMEs that are significantly associated with adverse overall survival. This distinctive enrichment of prognosis-associated RMEs motivated the prioritization of HCC for subsequent in-depth analyses. Guided by these observations, we applied machine-learning-based feature selection to derive an HCC-specific RME signature. We further characterized the intratumoral cellular distribution of these RMEs using single-cell RNA sequencing, enabling us to determine the immune and stromal compartments contributing to RME dysregulation. A LASSO-derived prognostic model was constructed and validated in multiple independent cohorts (TCGA-LIHC and GSE14520). Supportive functional assays on NAT10 were performed as representative evidence of biological relevance within the RME signature, rather than as definitive mechanistic validation.

Collectively, this integrative framework establishes a CNV-associated landscape of RME dysregulation in HCC and provides an association-based foundation for understanding how RNA modification programs relate to tumor progression and clinical outcome.

## 2 Results

### 2.1 Expression patterns of RNA modification enzymes across multiple cancer types

**Figure 1:**
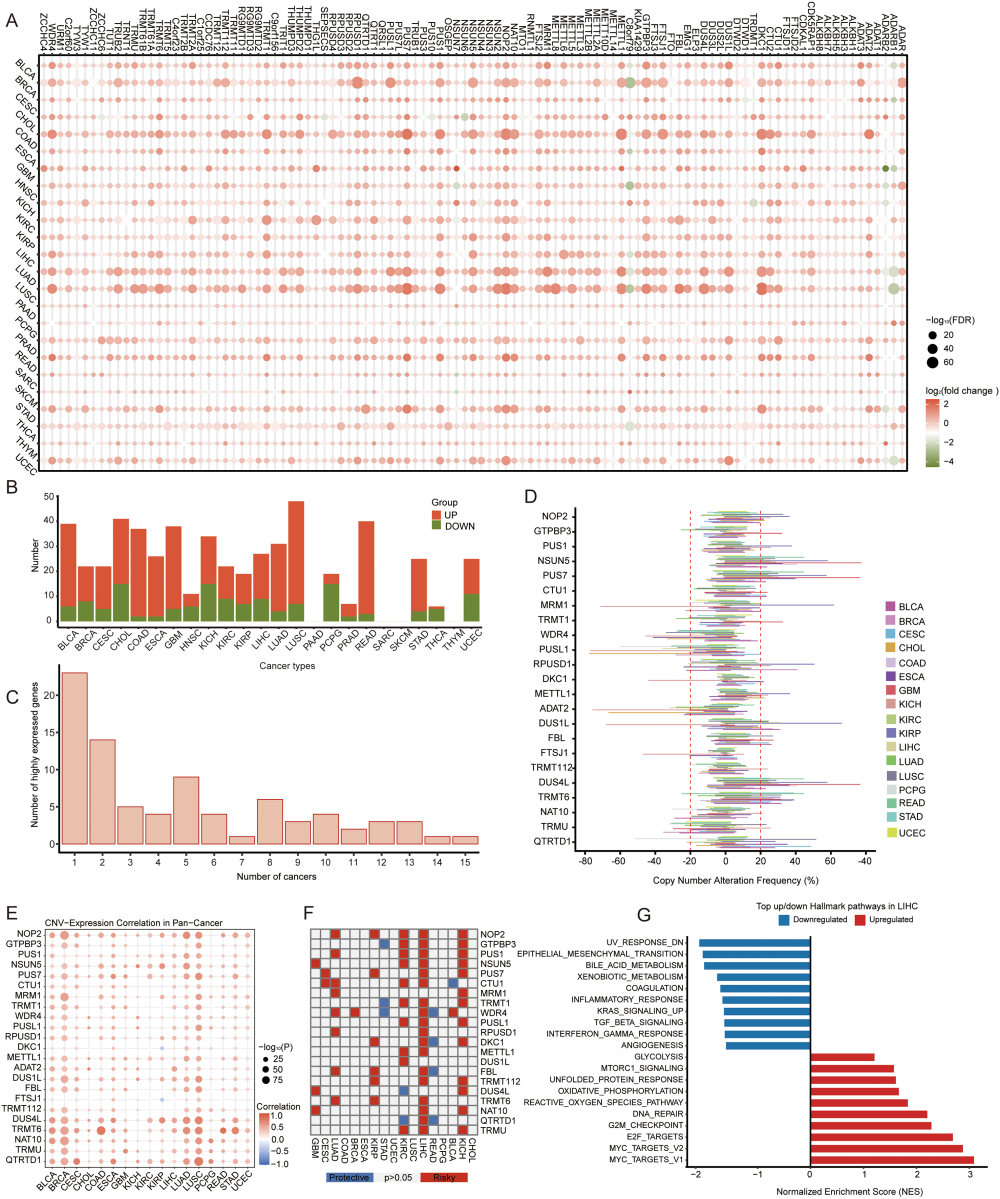
Expression patterns and prognostic significance of RNA modification enzyme genes (RMEs) across multiple cancer types. **(A)** Dot plot showing differential expression patterns of 105 RNA modification enzyme genes (RMEs) across 24 TCGA cancer types. Dot color represents log_2_ fold change (tumor vs. normal), and dot size corresponds to −log_10_(FDR). Significantly upregulated and downregulated genes are marked in red and green, respectively. **(B)** Bar plot summarizing the number of significantly upregulated and downregulated RME genes in each cancer type. Seven tumor types with fewer than ten dysregulated genes (<10% of the total) were excluded from downstream analyses. **(C)** The number of RME genes showing significant upregulation in each cancer. **(D)** Distribution of copy-number alteration (CNV) frequencies for 23 recurrently upregulated RME genes across 17 TCGA cancer types. **(E)** Spearman’s correlation between CNV and mRNA expression levels of RME genes. **(F)** Pan-cancer summary of associations between RME expression and overall survival across 17 TCGA cancer types. Each square represents one gene-cancer pair; red and blue denote higher expression associated with worse or better survival (*p* < 0.05), respectively. **(G)** Hallmark pathway analysis based on the RNA Modification Index (RMI) derived from ssGSEA in TCGA-LIHC.

As shown in **Supplementary Figure 1**, we compiled a total of 105 RME genes (**Supplementary Table 1**) from the RMBase v2.0, MODOMICS, and the Molecular Signatures Database (MSigDB) (12–14). Subsequently, we analyzed the 105 RME genes’ expression profiles in tumor and normal tissues from 24 TCGA cancer types (15, 16), including BLCA (Bladder Urothelial Carcinoma), BRCA (Breast Invasive Carcinoma), CESC (Cervical Squamous Cell Carcinoma and Endocervical Adenocarcinoma), CHOL (Cholangiocarcinoma), COAD (Colon Adenocarcinoma), ESCA (Esophageal Carcinoma), GBM (Glioblastoma Multiforme), HNSC (Head and Neck Squamous Cell Carcinoma), KICH (Kidney Chromophobe), KIRC (Kidney Renal Clear Cell Carcinoma), KIRP (Kidney Renal Papillary Cell Carcinoma), LIHC (Liver Hepatocellular Carcinoma), LUAD (Lung Adenocarcinoma), LUSC (Lung Squamous Cell Carcinoma), PAAD (Pancreatic Adenocarcinoma), PCPG (Pheochromocytoma and Paraganglioma), PRAD (Prostate Adenocarcinoma), READ (Rectum Adenocarcinoma), SARC (Sarcoma), SKCM (Skin Cutaneous Melanoma), STAD (Stomach Adenocarcinoma), THCA (Thyroid Carcinoma), THYM (Thymoma), UCEC (Uterine Corpus Endometrial Carcinoma). Our results showed that these RMEs were broadly upregulated across multiple cancers, with 17 cancer types showing predominantly upregulated RMEs and 3 cancer types showing predominantly downregulated RMEs, as exhibited in **Figures 1A, B**. For downstream comparative analyses, cancer types with fewer than 10 dysregulated genes (<10% of the total) were excluded, as such limited feature numbers are insufficient to support reliable enrichment and cross-cancer comparisons (**Figure 1B**). A total of 23 RME genes were recurrently upregulated in more than half of the 17 analyzed cancer types (**Supplementary Tables 2, 3**), including ADAT2, QTRTD1, FTSJ1, NSUN5, PUS7, DUS4L, NOP2, MRM1, DUS1L, FBL, CTU1, TRMT6, RPUSD1, DKC1, TRMT1, GTPBP3, METTL1, PUS1, WDR4, PUSL1, TRMT122, NAT10, and TRMU (**Figure 1C**).

### 2.2 CNV-associated expression patterns of RMEs across multiple cancer types

To investigate potential genomic factors associated with the widespread upregulation of RNA modification enzymes across human cancers, we next examined the relationship between RME expression patterns and somatic CNV, an important class of genomic alteration associated with transcriptional changes (17). Among the 23 RME genes that were recurrently upregulated in more than half of the analyzed cancer types, 22 exhibited significant positive associations between copy number gains and transcript abundance (**Figure 1E**; **Supplementary Table 4**). These 22 genes included ADAT2, CTU1, DKC1, DUS1L, DUS4L, FBL, FTSJ1, GTPBP3, METTL1, MRM1, NAT10, NOP2, NSUN5, PUS1, PUS7, PUSL1, QTRTD1, RPUSD1, TRMT1, TRMT6, TRMU, and WDR4.

The CNV-expression concordance of these RMEs was observed across multiple cancer types rather than being restricted to a single tumor entity. Several malignancies, including KIRP, GBM, LUSC, BLCA, ESCA, STAD, CESC, KIRC, LIHC, LUAD, COAD, and READ, harbored a relatively high number of RME genes whose expression levels were concordant with copy number gains (**Figure 1D**). These tumor types represent a subset of the 17 analyzed TCGA cohorts in which CNV-associated RME dysregulation was particularly prominent.

### 2.3 Clinical significance of recurrently upregulated RMEs with predominant representation in hepatocellular carcinoma

We further examined the clinical and functional relevance of these CNV-associated RMEs. Comparative survival analysis across multiple cancer types demonstrated that CNV-associated RMEs exhibit broad clinical relevance across various malignancies (**Figure 1F**). While different cancer types displayed RMEs associated with either increased or decreased overall survival, hepatocellular carcinoma showed a distinct pattern in which the majority of CNV-associated RMEs were significantly associated with poorer overall survival (**Figure 1F**). Specifically, 19 of the 22 RMEs were significantly associated with worse overall survival in HCC, representing the highest proportion of high-risk RMEs among all evaluated cancer types (**Supplementary Table 5**). This observation prompted us to prioritize HCC for subsequent analyses, aiming to further characterize the biological and clinical relevance of CNV-associated RMEs in a cancer type with significant prognostic enrichment.

To characterize transcriptomic programs associated with coordinated expression of RNA modification enzymes in hepatocellular carcinoma, we constructed an RNA Modification Index (RMI), defined as the ssGSEA enrichment score of the predefined RME gene set for each tumor sample. Based on the distribution of RMI values, samples were stratified into RMI-high and RMI-low groups by selecting the top and bottom 30% of tumors, respectively. To investigate transcriptomic differences associated with distinct RME expression states, we performed Hallmark pathway enrichment analysis comparing RMI-high versus RMI-low tumors (18, 19). RMI-high tumors showed significant enrichment of multiple tumor-associated transcriptional programs, encompassing pathways related to cell cycle regulation, metabolic reprogramming, and genomic stress responses. Representative enriched Hallmark pathways included MYC targets, E2F targets, G2M checkpoint, DNA repair, and mTORC1 signaling; whereas RMI-low tumors showed relative enrichment of inflammatory response, TGF-β signaling, and interferon-γ response gene sets (**Figure 1G**; **Supplementary Table 6**). Together, these analyses indicate that CNV-associated RME expression patterns in HCC correspond to distinct transcriptomic states with differential enrichment of tumor-associated and immune-related pathways.

### 2.4 Construction of an HCC-specific discriminative model using machine learning

To identify RMEs with robust tumor-discriminative capacity, we performed machine learning-based feature selection using the 19 RMEs that showed significant differential expression between HCC and adjacent normal tissues in the TCGA-LIHC cohort. A total of 87 algorithmic combinations, including penalized regression (Lasso, Ridge, Elastic Net), stepwise logistic regression, gradient boosting (GBM, glmBoost), support-vector machines (SVM), linear discriminant analysis (LDA), naive Bayes, and XGBoost were trained on the TCGA-LIHC dataset and evaluated using internal cross-validation and an external validation cohort (GSE25097).

**Figure 2:**
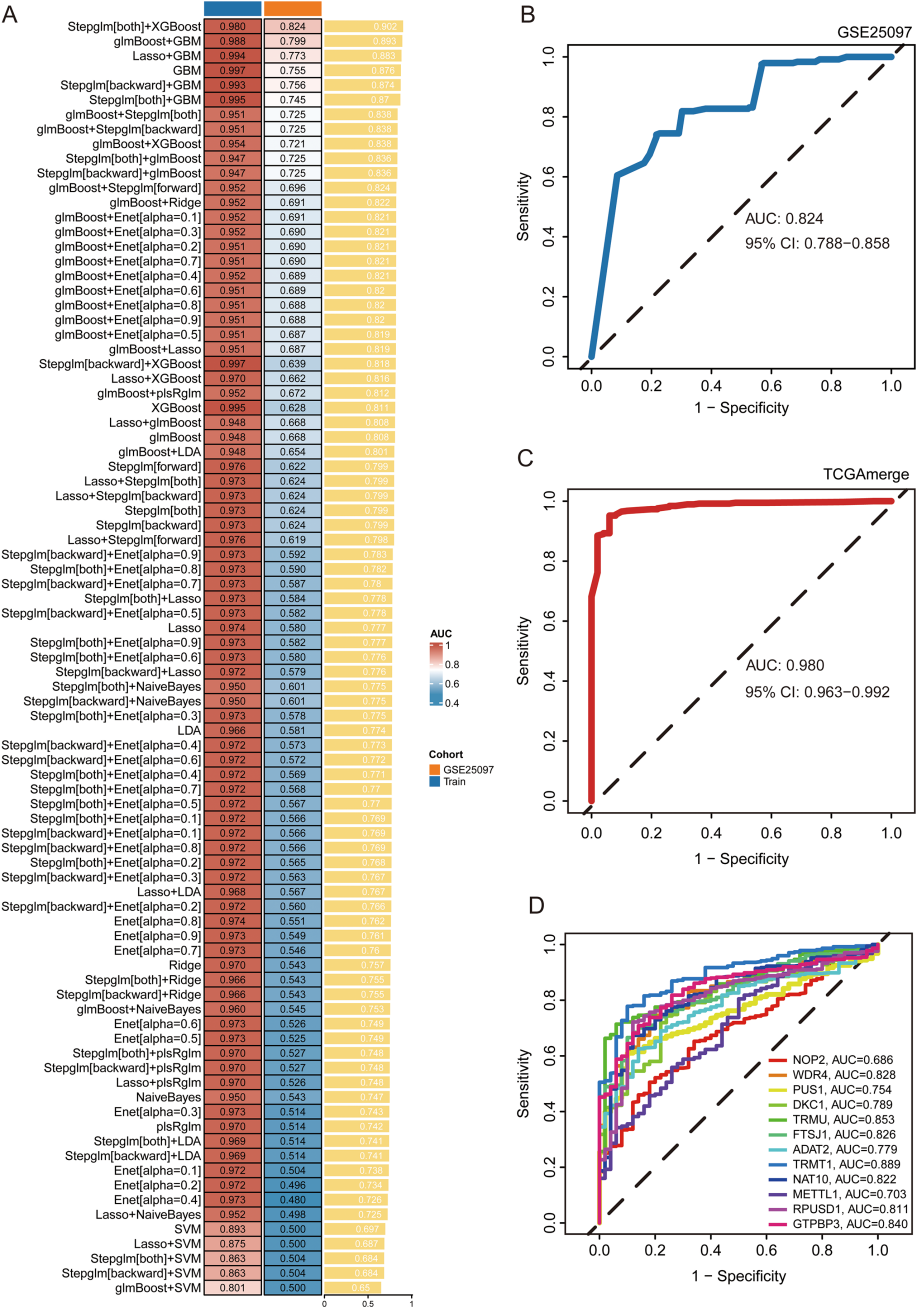
Performance evaluation of tumor-discriminative models and individual RMEs. **(A)** Heatmap summarizing the Area Under the Curve (AUC) values of multiple machine-learning model combinations for distinguishing HCC from normal tissues, evaluated in the training cohort and an independent validation cohort (GSE25097). **(B)** Receiver operating characteristic (ROC) curve of the diagnostic model in the external validation cohort (GSE25097). The area under the curve (AUC) and the corresponding 95% confidence interval (CI) are indicated. **(C)** ROC curve of the diagnostic model in the TCGA cohort. The AUC and its 95% confidence interval (CI) are shown. **(D)** ROC curves demonstrating the diagnostic performance of individual RMEs. Each colored curve represents a specific RME, with their corresponding AUC values listed in the legend.

Across all algorithms, the training cohort consistently demonstrated high discriminative ability (AUC range: 0.86-0.99), whereas substantial variability in model generalizability was observed in the GSE25097 cohort (AUC range: 0.50-0.83) (**Figure 2A**). Among the tested models, Stepglm[both] + XGBoost showed the best average performance across the training and external validation datasets (training AUC = 0.9799; GSE25097 cohort AUC = 0.8238) and demonstrating stable cross-cohort robustness (**Figures 2B, C**; **Supplementary Table 7**).

Feature selection from the optimal classifier yielded a non-redundant 12-gene RME panel for tumor–normal discrimination, comprising NOP2, WDR4, PUS1, DKC1, TRMU, FTSJ1, ADAT2, TRMT1, NAT10, METTL1, RPUSD1, and GTPBP3 (**Figure 2D**).

### 2.5 Single-cell analysis identifies T-cell-enriched expression of RMEs in HCC

**Figure 3:**
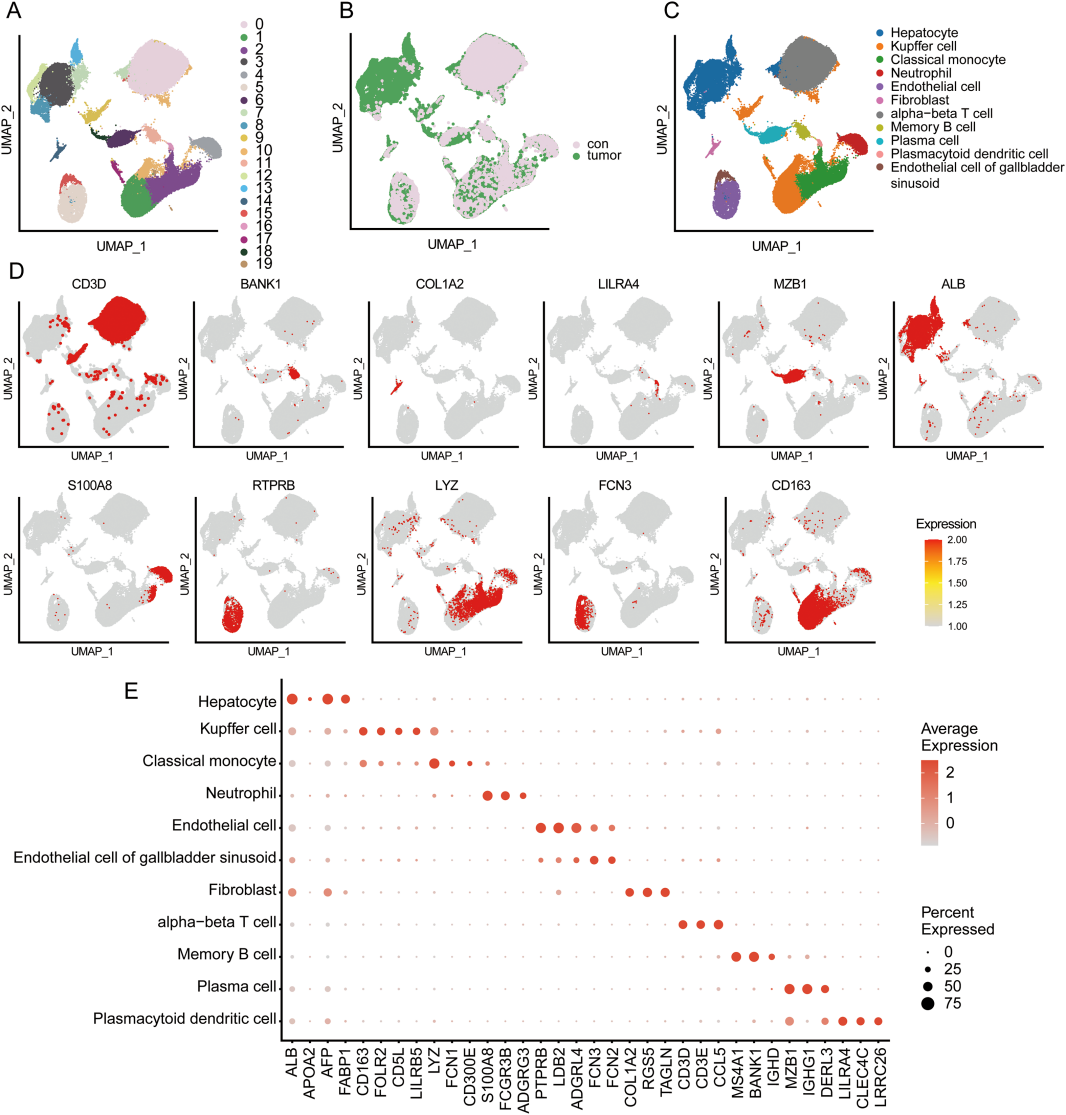
Single-cell transcriptomic atlas of hepatocellular carcinoma and adjacent normal liver tissue. **(A)** UMAP dimensionality reduction of integrated single-cell transcriptomes identified 20 transcriptionally distinct clusters (labeled 0-19). **(B)** Cells colored by sample origin demonstrate spatial distribution of tumor-derived (green) and adjacent normal tissue-derived (pink) cells across the UMAP embedding. **(C)** Manual annotation based on canonical marker gene expression resolved eleven cell types, including malignant hepatocytes, Kupffer cells, classical monocytes, neutrophils, endothelial cells, gallbladder-sinusoid-like endothelial cells, fibroblasts, α/β T cells, memory B cells, plasma cells, and plasmacytoid dendritic cells. **(D)** UMAP visualization depicting the spatial expression patterns of canonical marker genes across the single-cell landscape. Each panel displays an individual lineage marker, with color intensity representing normalized transcript abundance. **(E)** Dot plot showing the expression profiles of marker genes across the identified cell types. The size of each dot indicates the percentage of cells expressing the gene within that cell type (“Percent Expressed”), and the color intensity represents the average expression level (“Average Expression”).

To further investigate the biological context and cellular specificity of the 12 RNA modification enzymes identified through our machine-learning pipeline, we analyzed a publicly available single-cell RNA-sequencing dataset containing tumor and adjacent normal liver tissues (20). Following quality control to remove low-quality droplets and mitochondrial outliers, high-quality cells were retained and normalized for downstream analysis (**Supplementary Figure 2**) (21–24). Unsupervised clustering and dimensionality reduction identified eleven transcriptionally distinct cell populations (23, 25), including malignant hepatocytes, Kupffer cells, classical monocytes, neutrophils, endothelial cells, gallbladder-sinusoid-like endothelial cells, fibroblasts, α/β T cells, memory B cells, plasma cells, and plasmacytoid dendritic cells (**Figures 3A, C**). UMAP visualization demonstrated clear segregation of cells derived from tumor versus normal tissues (**Figure 3B**). Cell identities were annotated based on the expression of canonical lineage markers (26–29) including ALB/AFP/APOA2 for hepatocytes, CD163/FOLR2 for Kupffer cells, LYZ/FCN1 for monocytes, S100A8/FCGR3B for neutrophils, PECAM1/LDB2 for endothelial cells, FCN2/3 for gallbladder-sinusoid–like endothelial cells, COL1A2/RGS5 for fibroblasts, CD3D/3E/8A for α/β T cells, CLEC4C/LILRA4 for plasmacytoid dendritic cells, MS4A1/BANK1 for memory B cells, and MZB1/IGHG1 for plasma cells (**Figures 3D, E**).

**Figure 4:**
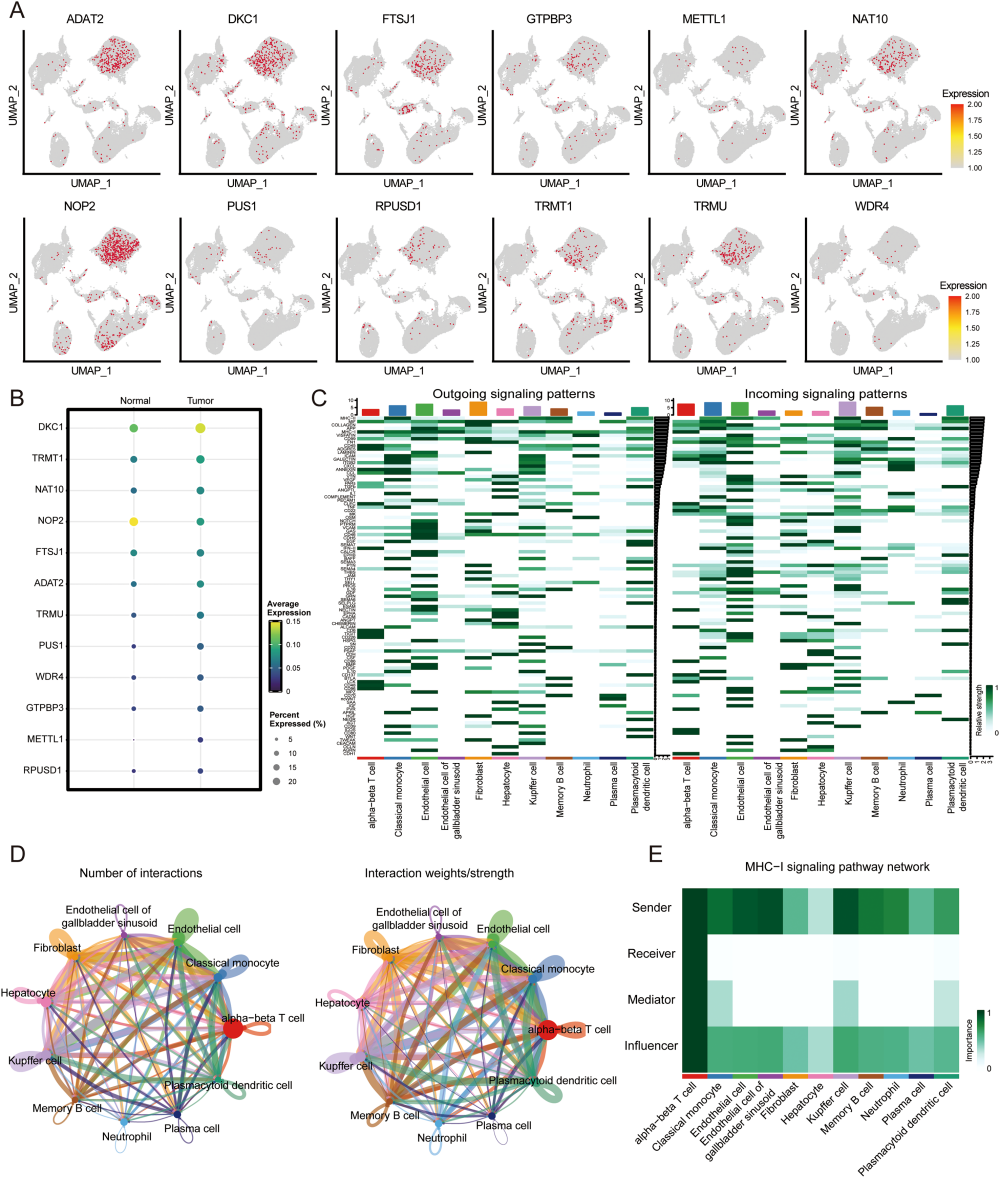
Single-cell dissection of RME expression heterogeneity and intercellular communication networks in HCC. **(A)** UMAP visualization depicting the spatial expression patterns of RMEs across the single-cell landscape. Each panel represents an individual RME, with color intensity reflecting normalized transcript abundance. **(B)** Comparative heatmap analysis of RME expression signatures between tumor (T) and matched adjacent normal (N) tissues across distinct cell populations. Color scale represents z-score normalized expression values. Statistical significance was determined by Wilcoxon rank-sum test. **(C)** Heatmap depicting outgoing (left) and incoming (right) signaling pathway patterns across identified cell populations. Color intensity represents relative signaling strength. **(D)** Circle plot showing the number of interactions and interaction weight/strength among different cell types. **(E)** Heatmap showing the roles of different cell types (sender, receiver, mediator, and influencer) in MHC-I signaling pathway network. Color intensity indicates relative importance scores.

Further dissection of the expression landscape revealed that the 12 RME-related genes exhibited two distinct expression patterns between tumor and adjacent normal tissues. (**Figure 4A**; **Supplementary Figure 3**). Among the 12 RMEs, NOP2 and FTSJ1 showed relatively lower expression levels in tumor-infiltrating cells compared with their counterparts in adjacent normal tissues at the single-cell level. In contrast, the remaining 10 RMEs, including PUS1, DKC1, WDR4, TRMU, TRMT1, NAT10, METTL1, RPUSD1, ADAT2, and GTPBP3, exhibited higher expression in tumor-infiltrating cells (**Figure 4B**; **Supplementary Table 8**). These single-cell patterns reflect cell-type-specific expression shifts and do not contradict the bulk-level differential expression used for the initial feature-selection step. Quantitatively, DKC1 demonstrated the strongest induction, showing both the highest fraction of expressing T cells and the greatest increase in average expression level within tumor T-cell clusters. NAT10, PUS1, TRMT1, TRMU, and ADAT2 also showed elevated expression, whereas the remaining RMEs displayed moderate but consistent elevation. Notably, these expression changes were predominantly confined to α/β T-cell clusters, with minimal alterations in macrophages, B cells or endothelial cells, suggesting preferential expression of selected RMEs within specific T-cell subsets in this dataset.

To further characterize intercellular communication patterns associated with T-cell populations exhibiting higher RME expression, we performed comprehensive cell-cell communication analysis using CellChat (30). Notably, cell-cell communication analysis revealed dense interaction networks among T cell subpopulations, with both the number and strength of signaling interactions being relatively high across these subsets (**Figure 4D**). Given our focus on T cell biology in the preceding analyses, we further examined the communication patterns of α/β T cells. Heatmap analysis of pathway-level signaling activity demonstrated that T cell subsets act as key mediators of intercellular communication in the tumor microenvironment, primarily signaling via MHC-I, CLEC, GALECTIN, CD86, NECTIN, PVR, CD137, ALCAM and LCK pathways. (**Figure 4C**; **Supplementary Figure 5**). We next focused on the MHC-I pathway, which showed the most prominent signaling activity in T cells. Remarkably, α/β T cells displayed significant roles across all four communication dimensions within the MHC-I signaling network, functioning as sender, receiver, mediator, and influencer (**Figure 4E**; **Supplementary Figure 6**). Collectively, these analyses indicate prominent MHC-I-mediated signaling involving α/β T cells within the inferred communication network.

### 2.6 Subpopulation-resolved analysis reveals preferential RME enrichment in regulatory T cells

**Figure 5:**
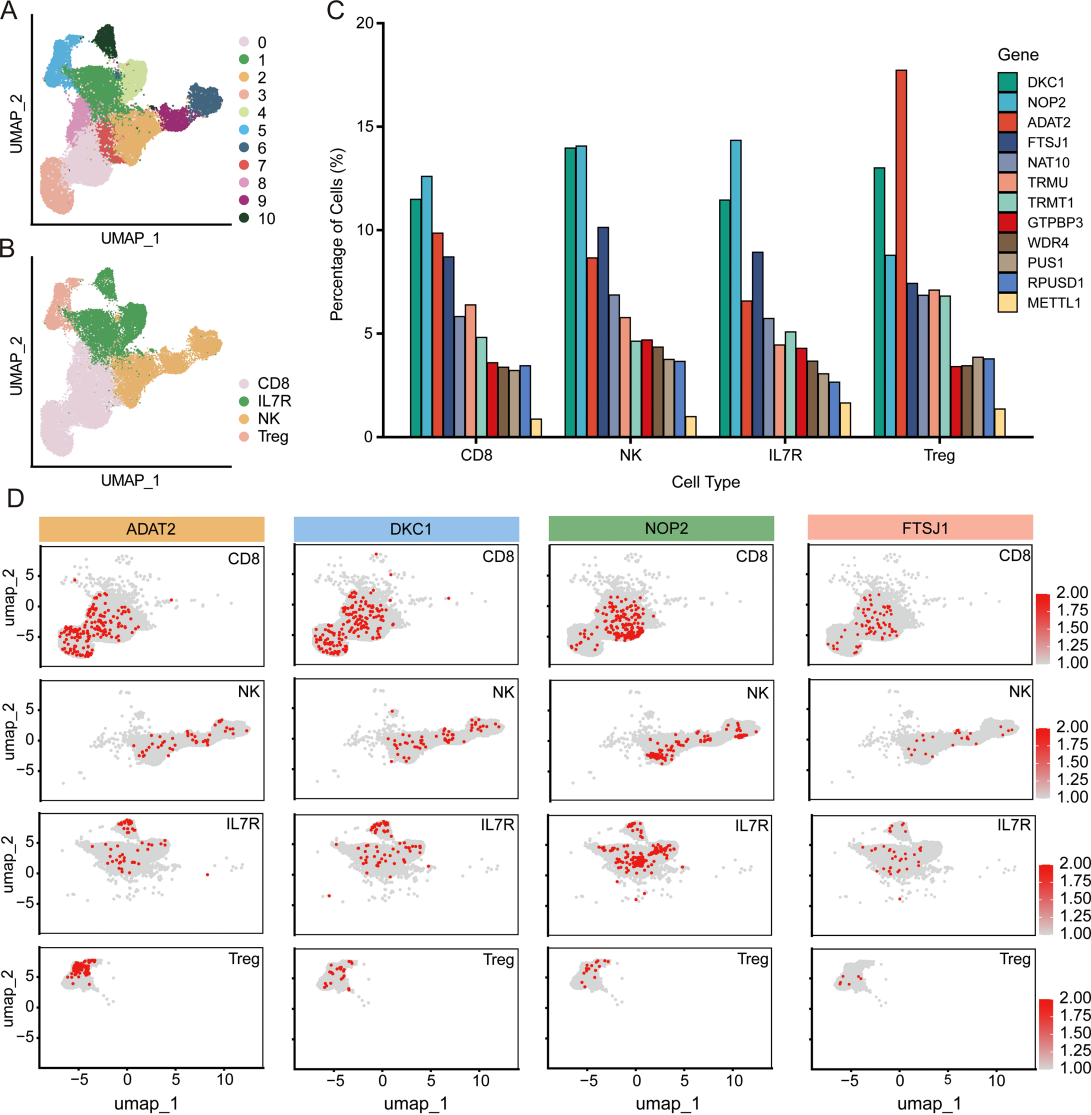
T cell subclustering analysis and subpopulation-specific expression of RMEs in HCC. **(A)** UMAP visualization of T cells derived from hepatocellular carcinoma samples, showing cell clusters identified by unsupervised clustering analysis. Each dot represents a single cell, colored by cluster identity. **(B)** UMAP feature plots displaying the expression of canonical marker genes used for annotation of major T cell subsets, including CD8^+^ cytotoxic T cells, IL7R^+^ memory/naïve T cells, regulatory T cells (Tregs), and natural killer (NK) cells. **(C)** Bar plot depicting subpopulation-associated expression patterns of 12 RMEs across the four T cell subsets (CD8^+^ T cells, NK cells, IL7R^+^ T cells, and Treg cells). Bar height represents the percentage of cells expressing each gene within the corresponding T cell subset. **(D)** Feature plots showing the cell-type–resolved expression distribution of representative RMEs (ADAT2, DKC1, NOP2, and FTSJ1) projected onto the UMAP embedding.

To gain deeper insights into the heterogeneous expression patterns of RNA modification enzymes within the T cell compartment, we performed refined subclustering analysis on tumor-infiltrating T lymphocytes. Unsupervised clustering identified four distinct T cell subpopulations based on canonical marker expression: CD8^+^ cytotoxic T cells, IL7R^+^ (CD127^+^) memory/naïve T cells, regulatory T cells (Tregs), and natural killer (NK) cells (**Figures 5A, B**) (31). UMAP visualization demonstrated clear spatial segregation among these subpopulations, with CD8^+^ T cells constituting the largest cluster, followed by IL7R^+^ T cells, NK cells, and Tregs. We first examined whether the overall expression landscape of the 12 prognostic RMEs differed between tumor-infiltrating and normal tissue-resident T cells. Intriguingly, dot plot analysis comparing tumor versus control samples revealed largely comparable expression patterns across all 12 RMEs at the bulk T cell population level. Both the percentage of expressing cells and average expression intensity demonstrated minimal differences between tumor and normal conditions for most genes, including NOP2, WDR4, PUS1, DKC1, TRMU, FTSJ1, ADAT2, TRMT1, NAT10, METTL1, RPUSD1, and GTPBP3 (**Supplementary Figure 4**). This observation suggests that the tumor microenvironment does not induce a global, uniform upregulation or downregulation of RMEs across the entire T cell population, but rather may exert subpopulation-specific regulatory effects.

In contrast to the relatively homogeneous expression observed at the global T cell level, subpopulation-resolved analysis unveiled striking heterogeneity in RME expression across the four T cell subsets (**Figure 5D**). Notably, ADAT2 exhibited the highest expression levels in regulatory T cells (Tregs), suggesting a potential association with regulatory T cell-specific transcriptional programs. (**Figure 5C**). Tregs are master regulators of immune homeostasis and play paradoxical roles in cancer, while essential for preventing autoimmunity, tumor-infiltrating Tregs often suppress anti-tumor immunity and correlate with poor prognosis in various malignancies. The elevated expression of ADAT2 in Tregs suggests that RNA modification pathways may be particularly active in this immunosuppressive population, potentially reflecting distinct transcriptional states associated with regulatory T cell identity within the tumor microenvironment.

### 2.7 Development and validation of an RME-based clinical prognostic model

**Figure 6:**
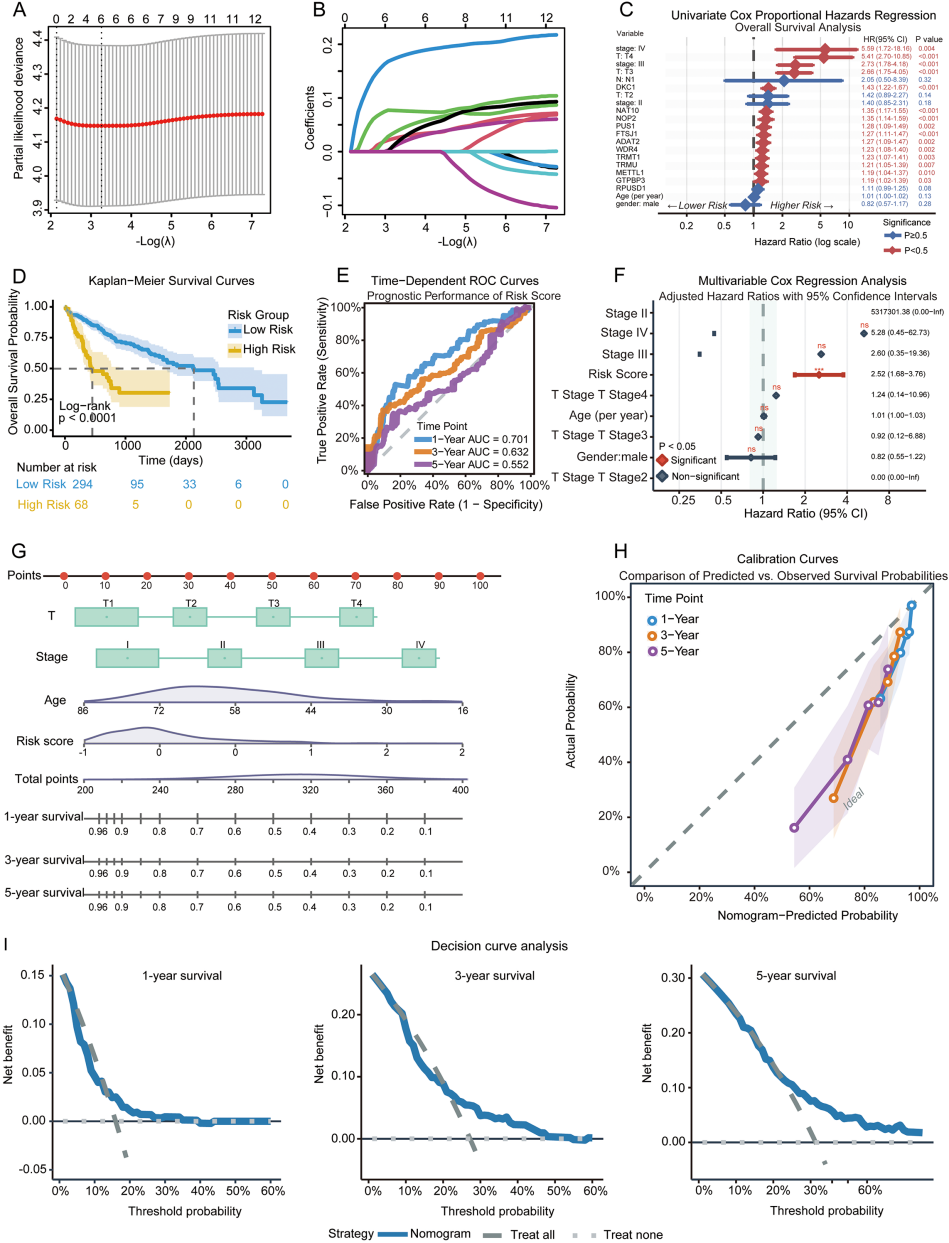
Construction of a risk score based on RNA modification enzyme genes. **(A)** LASSO regression analysis of the 12 prognostic-related genes. **(B)** Tenfold cross-validation used in the LASSO regression analysis. **(C)** Univariate Cox regression analysis and forest plot. **(D)** KM curves for high-risk and low-risk patients in the TCGA-LIHC. **(E)** ROC curves predicting 1-, 3-, and 5-year survival rates for patients in the TCGA-LIHC. **(F)** Multivariate analyses showing the correlation between survival rates, clinical parameters, and risk scores in TCGA-LIHC patients. **(G)** Establishment of the nomogram. **(H)** Calibration curves for nomograms. **(I)** Decision curves for 1-, 3-, and 5-year survival.

To translate the molecular alterations of RNA modification enzymes into clinically actionable predictors, we constructed a prognostic model in the TCGA-LIHC cohort using bulk RNA-seq data and corresponding survival outcomes. We first performed univariate Cox regression on the 12 RME genes derived from the machine-learning feature-selection pipeline. Eleven genes (DKC1, NAT10, NOP2, FTSJ1, WDR4, PUS1, ADAT2, TRMT1, TRMU, METTL1, and GTPBP3) exhibited significant adverse prognostic associations (all *p* < 0.05), highlighting their linkage to HCC progression and mortality (**Figure 6C**; **Supplementary Table 10**).

To reduce redundancy and account for collinearity among RMEs, LASSO-penalized Cox regression was applied (**Figures 6A, B**) (32), identifying a compact six-gene signature composed of DKC1, NAT10, PUS1, WDR4, ADAT2, and FTSJ1 (coefficient range: 0.011-0.174)(**Supplementary Table 9**). Subsequent multivariate Cox analysis confirmed that this six-gene panel independently predicted overall survival, even after adjusting for major clinical covariates including age, gender, tumor stage, and T stage (risk score HR = 2.52, 95% CI: 1.68-3.76, *p* = 6.89 × 10^−6^) (**Figure 6F**; **Supplementary Table 11**). A patient-specific risk score was then computed using the linear combination of normalized gene expression weighted by their LASSO coefficients, as shown in Equation 1:

(1) $$\begin{array}{c} Riskscore\;=\left(0.17\times DKC1\right)+\left(0.0767\times NAT10\right)+\left(0.0369\times PUS1\right)\\ +\left(0.0253\times WDR4\right)+\left(0.0149\times ADAT2\right)+\left(0.0113\times FTSJ1\right) \end{array}$$

Using the median risk score as the cutoff, we stratified patients into high- and low-risk groups. Kaplan-Meier analysis demonstrated a markedly worse survival outcome in the high-risk population (log-rank *p* < 0.0001) (**Figure 6D**; **Supplementary Table 12**). Time-dependent ROC curves showed that the RME-based model maintained reasonable discriminatory power at 1-year (AUC = 0.701), with moderate predictive ability at 3-year (AUC = 0.632) and 5-year (AUC = 0.552) (**Figure 6E**).

**Figure 7:**
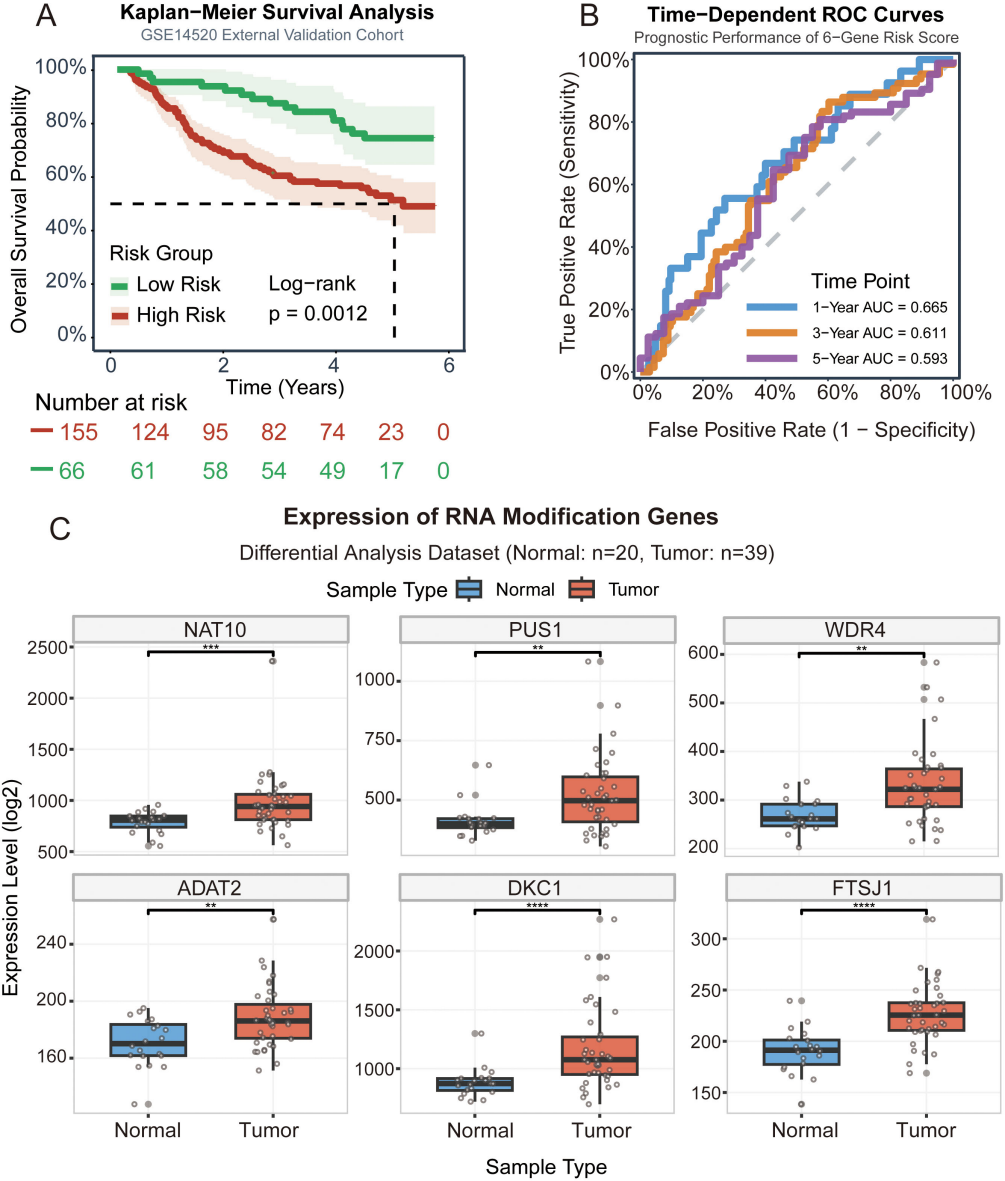
External validation of the prognostic model in HCC. **(A)** Kaplan-Meier survival curves showing overall survival of HCC patients stratified by risk score in the GSE14520 external validation cohort (n = 221). Patients were divided into high-risk (n = 155, red) and low-risk (n = 66, green) groups. Log-rank test *p* = 0.0012. **(B)** Time-dependent receiver operating characteristic (ROC) curves evaluating the predictive performance of the 6-gene risk score for 1-year (AUC = 0.665), 3-year (AUC = 0.611), and 5-year (AUC = 0.593) overall survival in the GSE14520 cohort. **(C)** Expression levels of RMEs in HCC tumor tissues (n = 39) compared to normal liver tissues (n = 20) from an independent GEO dataset. Statistical significance was determined by Wilcoxon rank-sum test. *p < 0.05, **p < 0.01, ***p < 0.001, ****p < 0.0001; ns, not significant.

To enhance clinical interpretability, we further integrated the RME-based risk score with conventional clinicopathological variables to construct a nomogram predicting 1-, 3-, and 5-year survival probabilities (**Figure 6G**). Calibration plots demonstrated excellent agreement between predicted and observed survival outcomes (**Figure 6H**), supporting the accuracy of the integrated model. Decision-curve analysis (DCA) additionally confirmed that the RME-based model provided greater net clinical benefit than treat-all or treat-none strategies across a broad range of threshold probabilities (**Figure 6I**), indicating clear practical utility in clinical decision-making. To further validate the robustness of our 6-gene prognostic model, we performed external validation using the GSE14520 cohort (n = 221). Patients were stratified into high-risk (n = 155) and low-risk (n = 66) groups based on the median risk score. Kaplan-Meier survival analysis demonstrated that patients in the high-risk group exhibited significantly poorer overall survival compared to those in the low-risk group (log-rank *p* = 0.0012, **Figure 7A**). Time-dependent ROC curve analysis revealed that the AUC values for predicting 1-, 3-, and 5-year overall survival were 0.665, 0.611, and 0.593, respectively (**Figure 7B**), indicating moderate predictive accuracy in the external cohort. Furthermore, we validated the expression patterns of the RNA modification-related genes using an independent GEO dataset (Normal: n = 20; Tumor: n = 39). Consistent with our findings in TCGA cohort, multiple signature genes including NAT10, DKC1, FTSJ1, PUS1, ADAT2 and WDR4 were upregulated in HCC tumor tissues compared to adjacent normal tissues (**Figure 7C**). Together, these findings establish the six-gene RME signature as an independent and clinically relevant prognostic indicator that complements conventional staging systems and offers improved risk stratification for patients with hepatocellular carcinoma.

### 2.8 Functional validation of the selected RMEs and pharmacogenomic response profiling

**Figure 8:**
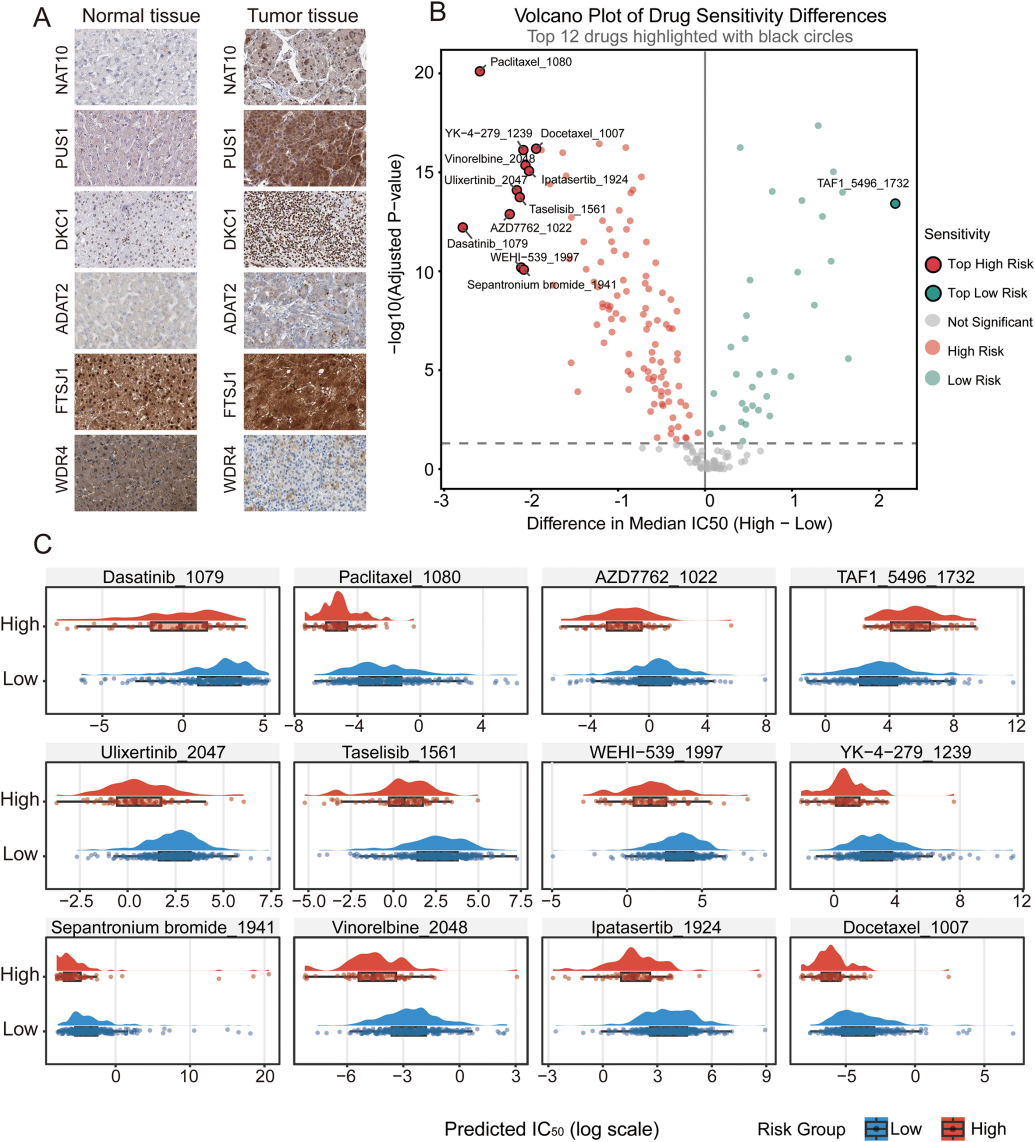
Protein-level validation of prognostic RMEs and drug sensitivity analysis in hepatocellular carcinoma. **(A)** Representative immunohistochemistry (IHC) images from the HPA database showing the expression of ADAT2, DKC1, FTSJ1, NAT10, PUS1, and WDR4 in normal liver tissues (left panel) and hepatocellular carcinoma tissues (right panel). Brown staining indicates positive expression. **(B)** Volcano plot illustrating the global landscape of predicted drug sensitivity differences across all 198 analyzed compounds. The x-axis represents the difference in median IC50 values (high-risk minus low-risk group), and the y-axis represents -log_10_(FDR). Each dot corresponds to a single drug. Colors indicate significance levels: red dots represent drugs to which the high-risk group is more sensitive (lower IC50), while blue dots represent drugs to which the low-risk group is more sensitive. The horizontal dashed line denotes the significance threshold; the vertical dashed line indicates zero difference. Representative drug names are labeled for highly significant compounds. **(C)** Rain cloud plots displaying the predicted IC50 values (log scale) of the top 12 most significantly differentiated drugs between high-risk (red) and low-risk (blue) groups. Each facet represents an individual drug (with drug ID shown in facet titles). The plots combine half-violin distributions with individual data points to illustrate the distribution of predicted drug responses in each risk group.

We performed validation experiments using independent clinical samples and publicly available datasets, to confirm the bioinformatic predictions at protein levels. Immunohistochemistry (IHC) images retrieved from the Human Protein Atlas (HPA) revealed that key RMEs (33), including NAT10, PUS1, DKC1 and ADAT2, exhibited markedly stronger nuclear and nucleolar staining in HCC tissues compared with adjacent normal liver tissues, consistent with their roles in RNA processing and ribosome biogenesis. In contrast, WDR4 showed reduced expression in tumor tissues, while FTSJ1 displayed no appreciable difference between tumor and normal counterparts (**Figure 8A**).

To investigate potential therapeutic implications of the RME-based stratification, we compared predicted drug sensitivity between the high-risk (n=68) and low-risk (n=294) groups based on the established risk score model (34). Wilcoxon rank-sum test with Benjamini-Hochberg correction revealed that 141 drugs (71.2%) exhibited significant sensitivity differences between the two groups (FDR< 0.05) (**Figure 8B**; **Supplementary Table 13**), indicating that RME-associated transcriptional states are linked to distinct pharmacologic vulnerabilities in hepatocellular carcinoma. We focused on the top 12 drugs ranked by absolute median IC50 difference (**Figure 8C**). Notably, 11 of these 12 compounds demonstrated preferential sensitivity in high-risk patients. Microtubule-targeting agents showed the most significant differential effects, with Paclitaxel (ΔIC50 = -2.59), Vinorelbine (ΔIC50 = -2.07), and Docetaxel (ΔIC50 = -1.94) exhibiting substantially lower IC50 values in high-risk tumors, consistent with their hyperproliferative phenotype. Kinase inhibitors targeting MAPK and PI3K/AKT pathways, including Ulixertinib (ΔIC50 = -2.16), Taselisib (ΔIC50 = -2.13), and Ipatasertib (ΔIC50 = -2.02), also favored high-risk patients, implicating oncogenic signaling dependencies in aggressive tumors. Additionally, agents targeting cell survival machinery such as AZD7762 (CHK1/2 inhibitor), Sepantronium bromide (survivin suppressant), and WEHI-539 (BCL-XL inhibitor) demonstrated enhanced efficacy in high-risk patients. In contrast, TAF1_5496, a TAF1 bromodomain inhibitor, was the sole compound showing preferential sensitivity in low-risk patients (ΔIC50 = +2.19), suggesting divergent epigenetic dependencies between risk strata. These findings provide a pharmacogenomic rationale for risk-adapted therapeutic strategies in HCC.

### 2.9 Supportive functional assessment of NAT10 in HCC

To provide limited biological context for the computational framework, we selected NAT10 as a representative RME for supportive functional assessment. This choice was based on its consistent inclusion across CNV, bulk transcriptomic, single-cell, and prognostic analyses, rather than on its relative effect size alone.

**Figure 9:**
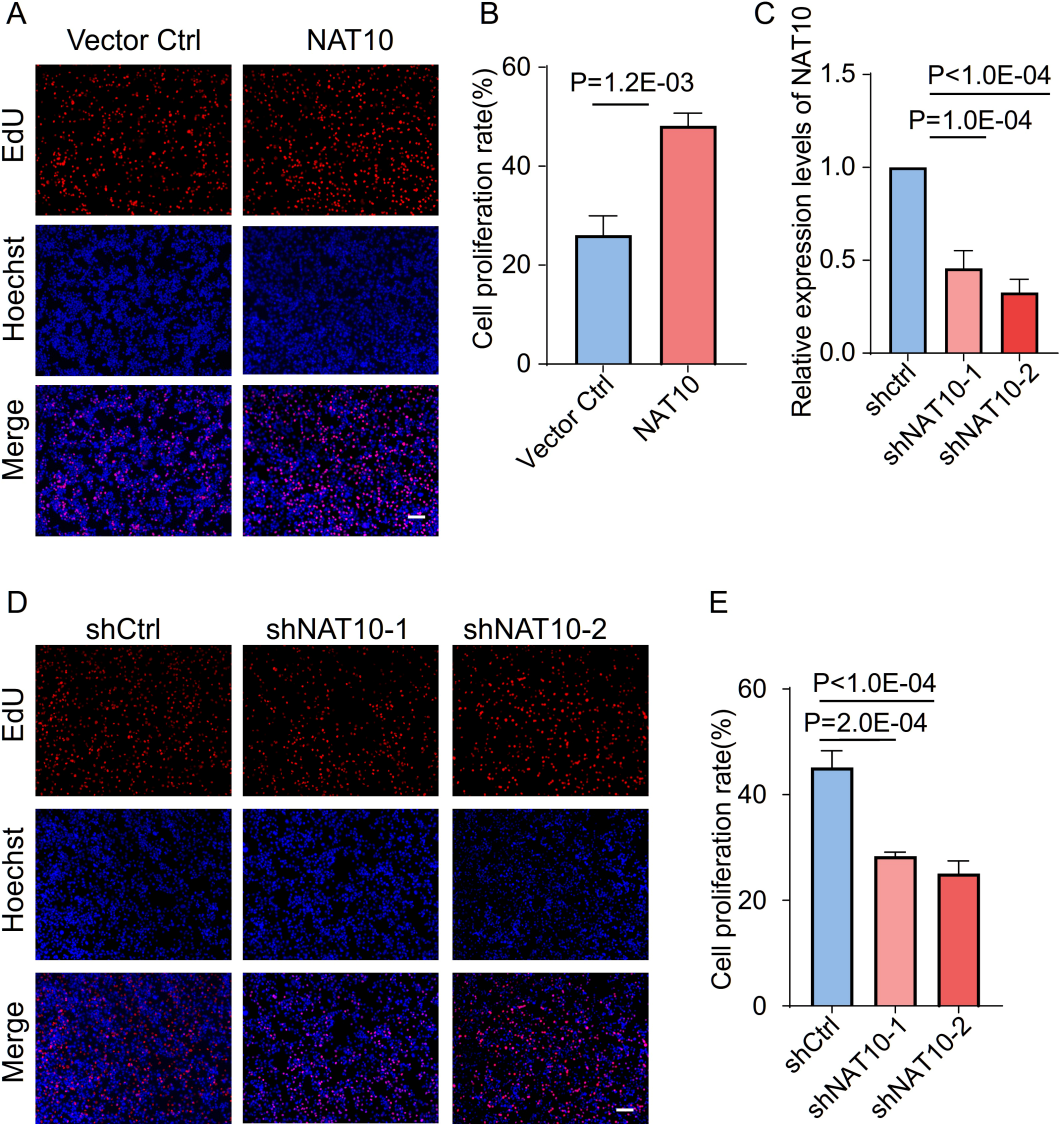
Supportive functional assessment of NAT10 in HepG2 cells. **(A)** Representative fluorescence images (left) and **(B)** quantification (right) of EdU incorporation assays in HepG2 cells transfected with NAT10 overexpression plasmid or vector control. Red: EdU-positive proliferating cells; Blue: Hoechst 33342 nuclear staining. Scale bar: 100 μm. Statistical analysis was performed using Student’s t-test. **(C)** qRT-PCR analysis of NAT10 knockdown efficiency using two independent shRNAs (shNAT10–1 and shNAT10-2) compared to control shRNA (shCtrl) in HepG2 cells. **(D)** Representative fluorescence images (left) and **(E)** quantification (right) of EdU incorporation assays in HepG2 cells transfected with shCtrl, shNAT10-1, or shNAT10-2. Red: EdU-positive proliferating cells; Blue: Hoechst 33342 nuclear staining. Scale bar: 100 μm. Statistical analysis was performed using one-way ANOVA with Dunnett’s multiple comparisons test. Data are presented as mean ± SD from three independent experiments.

To biologically validate the functional role of NAT10, we performed *in vitro* experiments using HepG2 cells. EdU incorporation assays showed that NAT10 overexpression was associated with increased proliferative activity in HepG2 cells compared with vector controls (*p* = 1.2E-03, **Figures 9A, B**). To further confirm these findings, we performed knockdown experiments using two independent shRNAs (**Supplementary Table 15**). qRT-PCR confirmed that both shNAT10–1 and shNAT10–2 effectively reduced NAT10 mRNA expression (**Figure 9C**; **Supplementary Table 16**). Conversely, knockdown of NAT10 using two independent shRNAs (shNAT10–1 and shNAT10-2) was associated with reduced proliferative activity in HepG2 cells compared with control cells (*p* = 2.0E-04 and *p* < 1.0E-04, respectively, **Figures 9D, E**). These data indicate a cell-intrinsic, pro-proliferative association of NAT10 in HepG2 cells under our experimental conditions, without implying a dominant or exclusive role among RMEs. This observation is provided as a limited, non-immune functional reference and does not address immune-related mechanisms, which remain to be investigated in future studies.

## 3 Discussion

This study systematically conducted an integrated analysis of RMEs across human cancers by combining single-cell RNA sequencing and bulk RNA sequencing data, with a particular focus on HCC. We demonstrated that RMEs are broadly upregulated in tumors, and this activation is strongly associated with copy-number amplification. Among all cancer types, HCC exhibits the highest proportion of RMEs associated with adverse clinical outcomes, highlighting a particularly coherent pattern of RME dysregulation in HCC. Based on these observations, we developed a machine-learning-derived 12-gene RME signature and established a prognostic model that demonstrated independent predictive value across cohorts and enabled individualized survival prediction when integrated with clinical staging.

The correlation between CNV gains and RME overexpression indicates that genomic amplification is associated with coordinated changes in RNA modification-related gene expression across cancers. Several components of the signature (e.g., DKC1, METTL1, and WDR4) have been previously reported to be associated with tumor cell proliferation, translational regulation, and metabolic adaptation in multiple cancer types, including hepatocellular carcinoma (35–40). The recovery of these well-characterized RMEs by our modeling framework supports the biological relevance of the signature, while highlighting additional, less-studied enzymes that warrant further investigation. Importantly, these contextual references are provided to indicate consistency with prior observations rather than to infer definitive mechanistic roles in the current study. In line with these previously reported associations, RME-high tumors in our cohort showed prominent enrichment of multiple tumor-associated transcriptional programs, including pathways related to cell cycle regulation, metabolic activity, and genomic stress responses, such as MYC targets, E2F signaling, G2M checkpoint regulation, DNA-damage repair, and mTORC1 activation. In contrast, RME-low tumors displayed marked activation of inflammatory response, Kras signaling, and TGF-β signaling. These associations suggest that tumors with higher RME expression are characterized by transcriptional programs related to proliferation and metabolic activity, whereas RME-low tumors show relative enrichment of inflammatory and immune-related signaling pathways.

Our single-cell analyses revealed cell-type-resolved expression heterogeneity of several RMEs within the HCC microenvironment, including relatively higher expression of selected enzymes in regulatory T cells compared with other T cell subsets. These findings are descriptive and association-based, and do not demonstrate direct immune regulatory mechanisms. Subpopulation-resolved profiling uncovered striking enrichment of ADAT2, DKC1, FTSJ1, and NOP2 in tumor-infiltrating Tregs—a finding of potential interest given that Treg accumulation has been reported to correlate with poor prognosis in HCC (41–43). Notably, ADAT2 showed comparatively higher expression in the Treg compartment in this dataset, suggesting that it may be prioritized for future mechanistic studies of immune-cell-specific RNA modification programs. ADAT2 has been reported to be associated with FOXP3-related transcriptional programs in other biological contexts; whether similar mechanisms operate in human HCC-associated Tregs remains to be determined (44). Concurrently, previous reports have linked DKC1 to cellular metabolic fitness in immune cells, raising the possibility that its preferential expression in Tregs may be associated with cellular metabolic states under tumor-associated stress conditions. However, these interpretations are speculative and require direct experimental validation (45, 46). Importantly, the preferential expression of selected RMEs in Tregs compared with CD8^+^ T cells highlights a potential area of interest for future studies, but no therapeutic conclusions can be drawn from the current data (47).

Although preferential RME expression in Tregs compared with CD8^+^ T cells may point to differential transcriptional states among T cell subsets, the current data do not support therapeutic inference. Any speculation regarding selective targeting of Treg-enriched RMEs or potential synergy with immune checkpoint blockade remains hypothetical and requires direct experimental validation (48, 49). Additionally, CellChat analysis showed that α/β T cells serve as important communication hubs in the HCC microenvironment, engaging in bidirectional crosstalk with antigen-presenting cells (such as Kupffer cells, classical monocytes, and plasmacytoid dendritic cells) through MHC-I-mediated signaling. The MHC-I pathway is crucial for cytotoxic CD8^+^T cell activation and antigen-specific immune responses (50). The dominant role of α/β T cells (especially CD8^+^T cells) in both sending and receiving intercellular signals suggests that transcriptional differences associated with RME expression in T cells may be linked to broader patterns of intercellular communication within the HCC immune microenvironment.

The 6-gene RME signature exhibited robust and reproducible prognostic value across independent cohorts and remained independently predictive after multivariable adjustment. Integrating the RME-based risk score with AJCC stage yielded a well-calibrated nomogram with significant clinical net benefit, highlighting its potential clinical applicability. Although several RMEs included in the signature have been explored as potential therapeutic targets in preclinical studies (51–54), the present work does not evaluate druggability or therapeutic efficacy. Accordingly, any therapeutic implications should be regarded as conceptual and hypothesis-generating.

Several limitations warrant acknowledgment. First, our single-cell dataset derives from a single cohort (GSE299340), necessitating validation in multi-center cohorts with diverse etiologies. Second, while IHC confirmed RME overexpression at the protein level, functional validation of immune-cell-specific roles of selected RMEs (such as ADAT2 in regulatory T cells) requires conditional knockout models or spatial transcriptomics to resolve cell-cell interaction dynamics. Third, drug sensitivity predictions are computational and require prospective clinical correlation. Future work will address these gaps through patient-derived organoid co-cultures and future translational studies stratified by RME signatures.

In summary, this study delineates a CNV-associated landscape of RME dysregulation in HCC and establishes an RME-based signature that is reproducibly associated with clinical outcome and immune-related transcriptomic features. By integrating pan-cancer genomics, machine learning-based modeling, and single-cell transcriptomic data, our work provides a coherent, association-based framework that supports further investigation of RNA modification programs in HCC.

## 4 Methods

### 4.1 Cell culture

The HepG2 cells were purchased from the American Type Culture Collection (ATCC). Cells were cultured in Dulbecco’s modified Eagle’s medium (DMEM) (Vivacell, Shanghai, China) supplemented with 10% fetal bovine serum (Vivacell, Shanghai, China) and 1% penicillin-streptomycin (WISENT Inc., CA). The HepG2 cells were cultured under a humidified atmosphere of 5% CO2 at 37°C.

### 4.2 Plasmid construction and cell transfection

The full-length coding sequence of human *NAT10* was cloned into the pcDNA3.1-3×FLAG vector to generate the NAT10 overexpression plasmid. The empty pcDNA3.1-3×FLAG vector was used as a negative control. The primers used for cloning are listed in **Supplementary Table 14**. For NAT10 knockdown, short hairpin RNA (shRNA) targeting *NAT10* was cloned into the pLKO.1 vector. A non-targeting scramble shRNA was used as a negative control. The shRNA target sequences are listed in **Supplementary Table 15**. For cell transfection, HepG2 cells were seeded into 6-well plates and cultured to 70-80% confluence. Plasmids were transfected into cells using Lipofectamine 3000 reagent (Invitrogen, USA) according to the manufacturer’s instructions. Cells were harvested at 48 h post-transfection for subsequent experiments.

### 4.3 RNA isolation and reverse transcription-quantitative PCR

Total RNA was isolated from cell samples using Trizol Reagent (Ambion, USA) according to the manufacturer’s instructions. RNA concentration and purity were measured using a One Drop^®^ OD-1000 Spectrophotometer (Nanjing Wuyi Corporation, China). Reverse transcription was performed using the HiScript II 1st Strand cDNA Synthesis Kit (Vazyme, China) following the manufacturer’s protocol. The following quantitative PCR (qPCR) analysis was conducted using SYBR^®^ Green Master Mix (Vazyme, China) on a Light Cycler^®^ 96 system (Roche, USA). GAPDH was used as the internal control for normalization. The primers used for the real-time PCR are listed in **Supplementary Table 16**. Relative fold changes in gene expression were calculated using the 2^−ΔΔCt^ method.

### 4.4 Cell proliferation assay

Cell proliferation was assessed using the Cell-Light EdU Apollo 567 *In Vitro* Kit (RiboBio, China) according to the manufacturer’s instructions. Briefly, transfected HepG2 cells were seeded into 96-well plates at a density of 5 × 10³ cells per well. After 24 h of culture, cells were incubated with 50 μM EdU reagent for 2 h at 37 °C. Subsequently, cells were fixed with 4% paraformaldehyde for 30 min, permeabilized with 0.5% Triton X-100 for 10 min, and stained with Apollo fluorescent dye for 30 min. Nuclei were counterstained with Hoechst 33342 for 30 min. Images were captured using a fluorescence microscope, and the percentage of EdU-positive cells was calculated from three random fields per group.

### 4.5 Datasets and sources

Transcriptomic, genomic, and epigenomic datasets were obtained from multiple public repositories. mRNA expression profiles, copy number alterations, and clinical data for 17 cancer types with matched tumor and normal tissues-including BLCA, BRCA, CESC, CHOL, COAD, ESCA, GBM, KICH, KIRC, KIRP, LIHC, LUAD, LUSC, PCPG, READ, STAD and UCEC were downloaded from the Broad GDAC Firehose portal (http://gdac.broadinstitute.org).

### 4.6 Differential expression analysis

A total of 105 RNA modification enzyme (RME) genes were curated from RMBase v2.0, MODOMICS, and the Molecular Signatures Database (MSigDB). Differential expression between tumor and normal tissues across 24 cancer types was analyzed using the limma R package. Genes with fold change ≥ 1.5 and an adjusted *p*-value< 0.05 (Benjamini-Hochberg correction) were considered significantly dysregulated.

### 4.7 Copy number variation analysis

CNV data generated by GISTIC2.0 (55), which enables sensitive and confident localization of recurrent copy-number alterations in human cancers, were obtained from TCGA and analyzed to identify CNV-associated genes across pan-cancer cohorts. Spearman rank correlations between CNV levels and corresponding gene expression levels were computed using R (v4.3.1).

A gene was classified as CNV-associated if it satisfied all of the following four criteria:

1. CNV frequency threshold: ≥ 20% of tumor samples exhibited CNV gain or loss events (|CNV| > 0.3), representing high-frequency genomic alterations based on GISTIC2.0 standard thresholds;
2. Population-level effect threshold: mean CNV level across tumor samples ≥ 0.05, ensuring a detectable population-level copy number effect;
3. CNV-expression correlation threshold: Spearman correlation coefficient between CNV and gene expression ≥ 0.3, indicating at least moderate positive correlation;
4. Statistical significance threshold: The correlation had a *p* < 0.05 after FDR correction for multiple testing, using the Benjamini-Hochberg method.

This quadruple filtering strategy ensured the identification of genes whose expression is robustly driven by copy number alterations at both individual and population levels.

### 4.8 RNA modification index and pathway enrichment analysis

RMI was designed to represent global RNA-modification activity. RMI was calculated as a single-sample gene set enrichment score of the RNA modification enzyme (RME) gene set using single-sample gene set enrichment analysis (ssGSEA) implemented in the GSVA R package. Samples were stratified into top and bottom 30% based on RMI, and hallmark pathway enrichment was assessed using Gene Set Enrichment Analysis (GSEA) from MSigDB.

### 4.9 Machine-learning modeling

Batch-corrected, log_2_-normalized expression matrices from TCGA-LIHC and the external validation cohort GSE25097 were harmonized using shared genes, and batch effects were adjusted using ComBat (sva package). Samples from the TCGA merged dataset were defined as the training cohort, and the remaining cohorts served as independent test sets. Tumor vs normal status was encoded as a binary outcome, and gene expression values were z-score-standardized within cohort.

A predefined panel of machine-learning algorithms was implemented in R, including penalized logistic regression (Lasso, Ridge, Elastic Net; glmnet), stepwise generalized linear models (Stepglm), support-vector machines (SVM), linear discriminant analysis (LDA), Naive Bayes, gradient boosting machines (GBM), glmBoost, partial least squares-GLM (plsRglm), random forests, and XGBoost. Most models were applied using a two-step scheme, in which an initial feature-selection step (e.g., Lasso, Elastic Net, glmBoost, random forest, GBM, XGBoost) was used to identify informative genes, followed by refitting of the designated classifier on the reduced feature set. Models retaining ≤ 5 genes were excluded from downstream analyses. For each model, continuous prediction scores were generated in the training and test cohorts, and the area under the receiver operating characteristic curve (AUC) was calculated using the pROC package. Model performance across cohorts was summarized using heatmap visualization (ComplexHeatmap). Model selection was based on the average AUC across training and validation cohorts, and the genes retained in the selected model were used for downstream biological analyses and prognostic modeling.

### 4.10 Nomogram construction and validation

A prognostic nomogram integrating the RME risk score with clinical variables (TNM stage, age, gender) was constructed using the rms R package. Calibration curves (1,000 bootstrap resamples) assessed predictive accuracy, while decision-curve analysis (rmda package) evaluated clinical net benefit. External validation was performed in the GSE14520 cohort.

### 4.11 Single-cell RNA-seq data processing and analysis

Publicly available scRNA-seq data of HCC and adjacent normal liver tissues were obtained from the Gene Expression Omnibus (GEO) database (accession number: GSE299340). Raw count matrices were processed and analyzed using Seurat (v5.0.3) in R.

### 4.12 Quality control and normalization

Cells were filtered using the following criteria:

- fewer than 300 detected genes were removed;
- genes expressed in fewer than 5 cells were excluded;
- cells with< 20% mitochondrial transcripts, > 3% ribosomal content, and< 1% erythrocyte transcripts were retained.

High-quality cells passing QC were normalized with the LogNormalize method and scaled to 10,000 transcripts per cell. A total of 2000 highly variable genes were identified and used for downstream analysis, while low-quality droplets and mitochondrial outliers were excluded to minimize technical noise.

### 4.13 Dimensionality reduction, batch correction, and clustering

Highly variable genes were selected for principal component analysis (PCA). The top 30 principal components were used for downstream analyses. Batch effects among samples were corrected using the Harmony algorithm. Dimensionality reduction was performed using Uniform Manifold Approximation and Projection (UMAP) and t-distributed Stochastic Neighbor Embedding (t-SNE). Unsupervised graph-based clustering was applied using the FindNeighbors and FindClusters functions in Seurat with a resolution of 0.5, which identified 20 transcriptionally distinct clusters.

### 4.14 Cell-type annotation

Cell-type identities were assigned based on canonical marker genes and manual inspection of marker expression patterns. Specifically, ALB/AFP/APOA2 defined hepatocytes, CD68/CD163 defined macrophages (Kupffer cells), LYZ/FCN1 defined monocytes, S100A8/S100A9 defined neutrophils, PECAM1/VWF defined endothelial cells, COL1A1/COL1A2 defined fibroblasts, CD3D/CD3E/CD8A defined T cells, and CD79A/IGHG1 defined B cells. The final annotation included eleven major populations: malignant hepatocytes, Kupffer cells, classical monocytes, neutrophils, endothelial cells, gallbladder-sinusoid-like endothelial cells, fibroblasts, α/β T cells, memory B cells, plasma cells, and plasmacytoid dendritic cells.

### 4.15 Identification of RNA modification enzyme expression patterns

Expression profiles of 12 RNA modification enzyme (RME) genes (NOP2, WDR4, PUS1, DKC1, TRMU, FTSJ1, ADAT2, TRMT1, NAT10, METTL1, RPUSD1 and GTPBP3) were extracted from the integrated dataset. The expression levels of these genes were visualized using FeaturePlot, DotPlot, and VlnPlot functions in Seurat. Dot plots were used to summarize the average expression and percentage of expressing cells across each cell type, whereas violin plots compared gene expression levels between tumor and adjacent normal samples.

### 4.16 Marker gene identification and visualization

Cluster-specific marker genes were identified using the FindAllMarkers function with the Wilcoxon rank-sum test, using thresholds of |log_2_ fold change| ≥ 0.25, adjusted *p*-value< 0.05, and min.pct = 0.2. Heatmaps and dot plots were generated to visualize the expression of top marker genes in each cluster.

### 4.17 Cell-cell interaction analysis

To investigate intercellular communication among distinct hepatic cell populations, we performed a comprehensive ligand-receptor interaction analysis using CellChat (v1.6.1) with the human CellChatDB database (v1.x). Significant interactions were determined via a permutation test (*p* < 0.05). Visualization of global communication networks, signaling roles, and interaction strength was generated using the netVisual and netAnalysis functions within CellChat, along with the ggplot2 package in R.

### 4.18 Statistical analysis and data visualization

All statistical analyses were conducted in R (v4.3). Figures were generated using ggplot2, cowplot, and patchwork. The final cell-type maps and RME expression landscapes were visualized on UMAP coordinates. IHC images of RME proteins were obtained from the Human Protein Atlas (HPA).

### 4.19 Drug sensitivity analysis

Drug sensitivity prediction was performed on TCGA-LIHC samples using the oncoPredict algorithm. This method trains Ridge regression models based on drug screening data and gene expression profiles from the Genomics of Drug Sensitivity in Cancer 2 (GDSC2) database to predict drug response in clinical samples. Batch effect correction between TCGA and GDSC data was performed using the ComBat algorithm. Genes with low variance (bottom 20th percentile) were removed, and 17,077 genes common to both datasets were retained for analysis. The calcPhenotype function was used to predict IC50 values for 198 antitumor compounds across 413 TCGA-LIHC samples, with lower IC50 values indicating greater drug sensitivity.

### 4.20 Statistical analysis

All analyses were conducted using R (v4.3.1) and GraphPad Prism (v9).

Continuous variables were compared using the Student’s t-test for two-group comparisons or one-way analysis of variance (ANOVA) followed by Dunnett’s *post hoc* test for multiple-group comparisons. Non-parametric comparisons were performed using the Wilcoxon rank-sum test. Survival differences were evaluated by Kaplan-Meier analysis with the log-rank test. Cox proportional hazards models were used to estimate hazard ratios (HRs) and 95% CIs. Correlations were assessed using Pearson or Spearman coefficients. Two-sided *p*-values< 0.05 were considered statistically significant, with false discovery rate (FDR) correction applied where applicable.

## Data Availability

The original contributions presented in the study are included in the article/**Supplementary Material**. Further inquiries can be directed to the corresponding authors.
