# Supplementary material for: Multi-omics characterization of RNA modification enzymes identifies NAT10 as a functionally validated prognostic biomarker in hepatocellular carcinoma
Source: Front Immunol. 2026 Jan 28;17:1764106. doi: 10.3389/fimmu.2026.1764106 (PMC12891137; doi:10.3389/fimmu.2026.1764106)
Supplement: Supplementary file 1 [file DataSheet1.pdf]

Supplementary Material

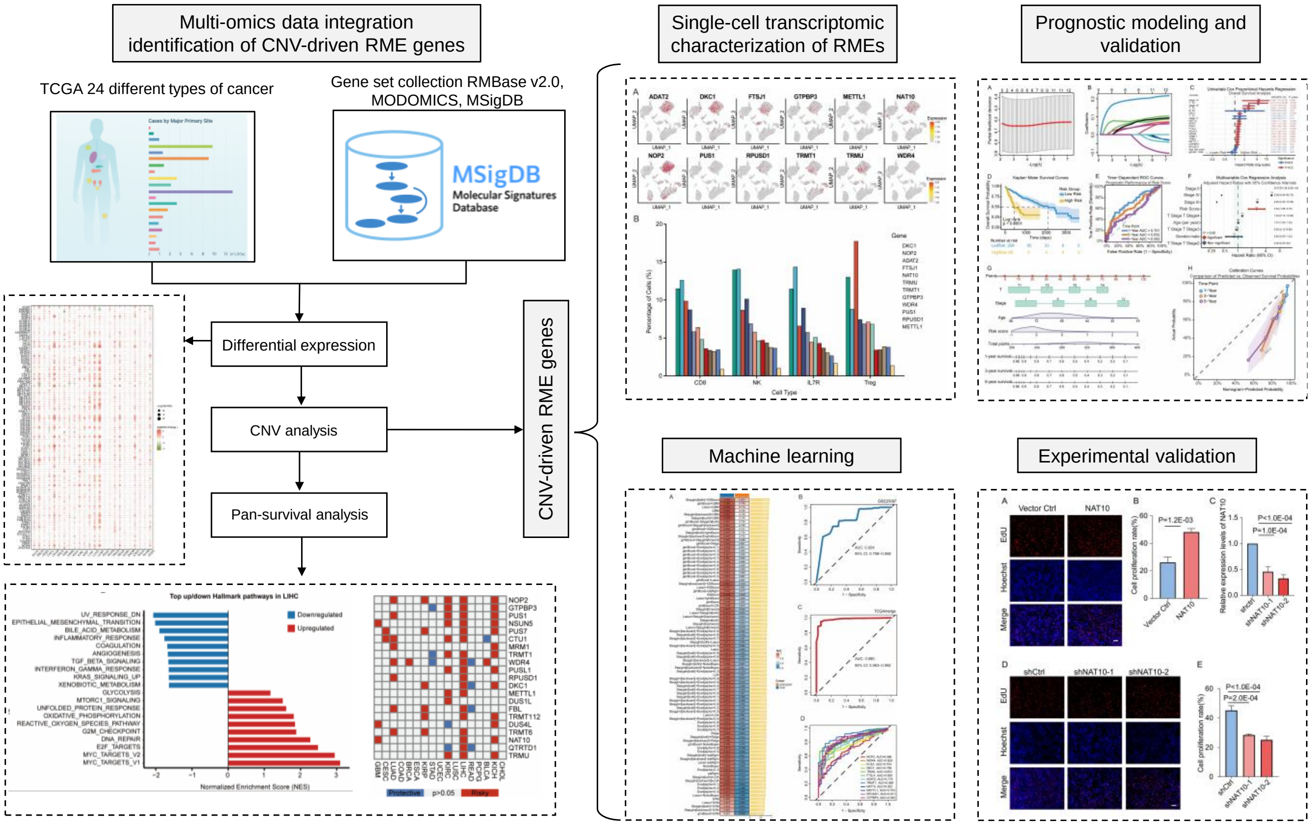

Supplementary Figure 1. Workflow used to identify CNV-driven RNA modification enzyme (RME) genes and characterize their immunological and clinical significance in hepatocellular carcinoma.

Multi-omics integration across TCGA pan-cancer cohorts and curated gene sets (RMBase v2.0, MODOMICS, MSigDB) was first performed to determine differentially expressed RMEs, copy-number alterations, and pan-cancer survival associations. The resulting CNV-driven RME genes were next analyzed using single-cell RNA-seq data to define their cell type-specific expression patterns within the tumor immune microenvironment. Machine-learning algorithms were applied to construct a robust RME-based diagnostic signature, followed by independent validation across multiple cohorts. Finally, experimental immunohistochemistry analyses were conducted to verify the protein-level expression of key RMEs in paired normal and tumor tissues. Together, this workflow summarizes the multi-layered computational and experimental approaches used throughout the study.

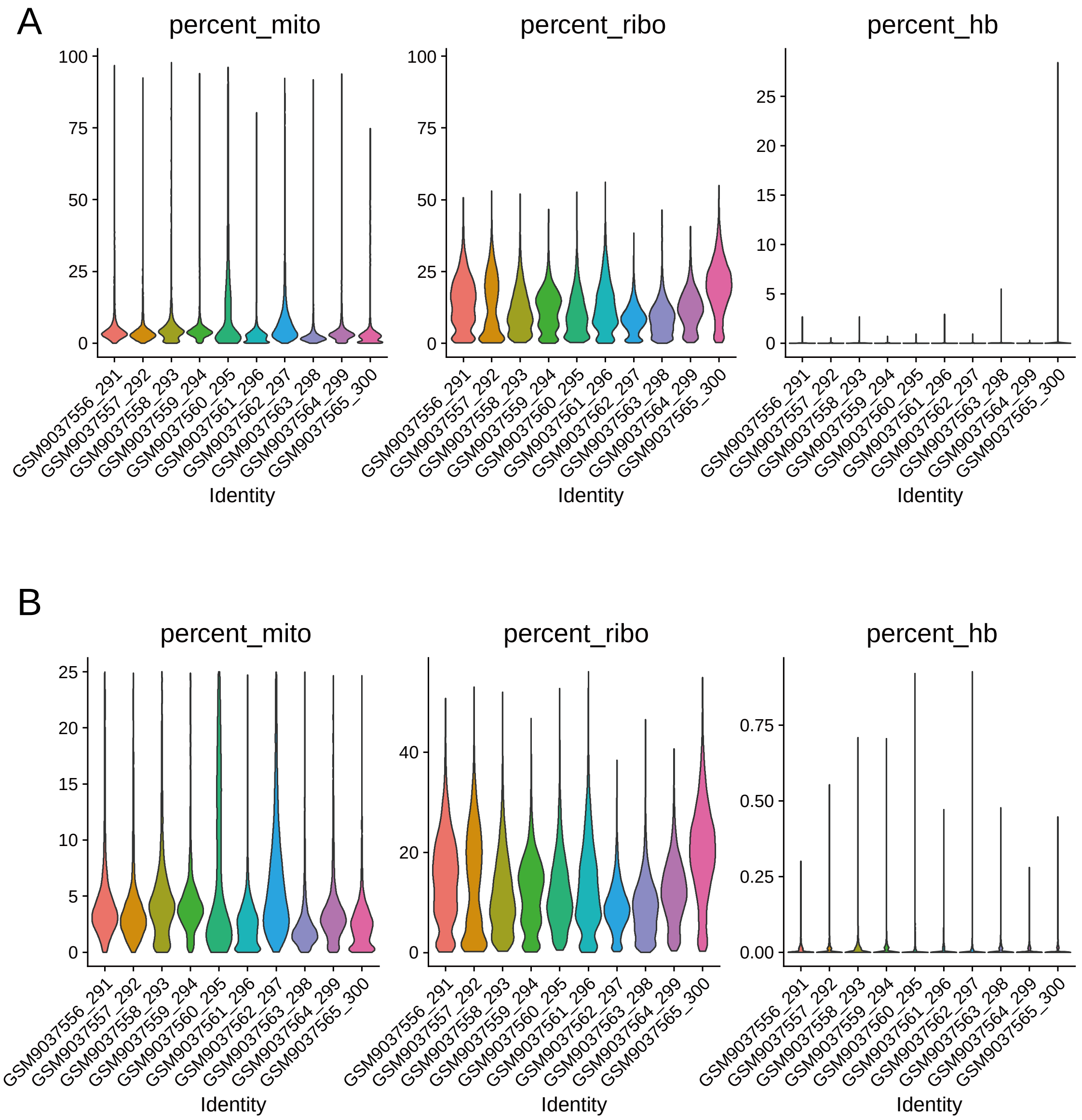

Supplementary Figure 2. Quality control metrics before and after cell filtering.

Violin plots showing the distribution of mitochondrial (percent_mito), ribosomal (percent_ribo), and hemoglobin (percent_hb) gene percentages across samples. (A) Before filtering, cells exhibited variable quality metrics including high mitochondrial content indicative of stressed/dying cells and detectable erythrocyte contamination. (B) After filtering (retaining cells with < 20% mitochondrial transcripts, >3% ribosomal content, and < 1% hemoglobin transcripts), the dataset showed uniform distributions with reduced mitochondrial percentages, adequate ribosomal content, and minimal red blood cell contamination, confirming successful quality control with minimal batch effects.

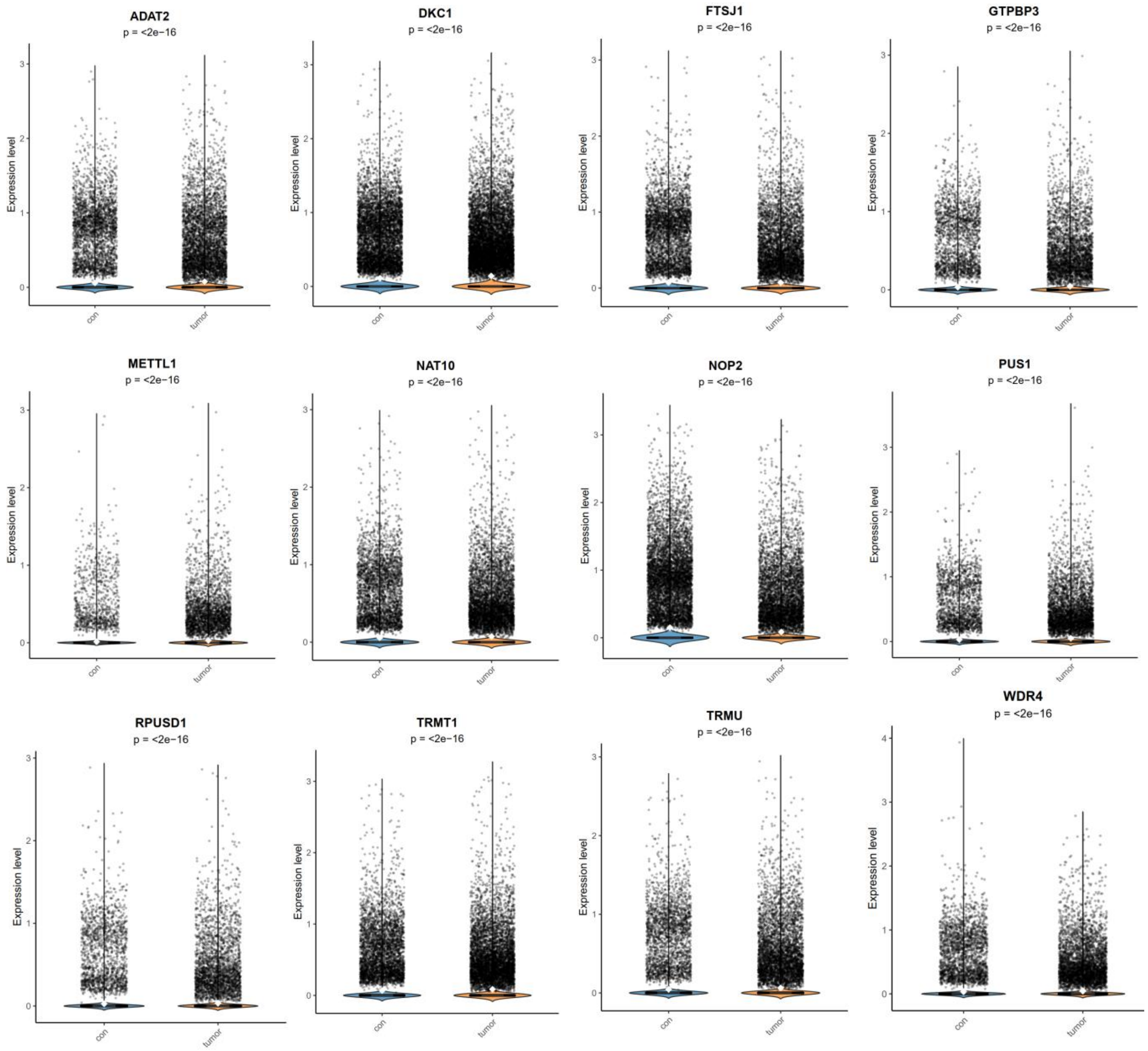

Supplementary Figure 3. Expression profiles of RMEs between tumor and adjacent normal tissues.

Visualization of expression patterns for 12 RMEs (NOP2, WDR4, PUS1, DKC1, TRMU, FTSJ1, ADAT2, TRMT1, NAT10, METTL1, RPUSD1, and GTPBP3) between tumor and adjacent normal tissues.

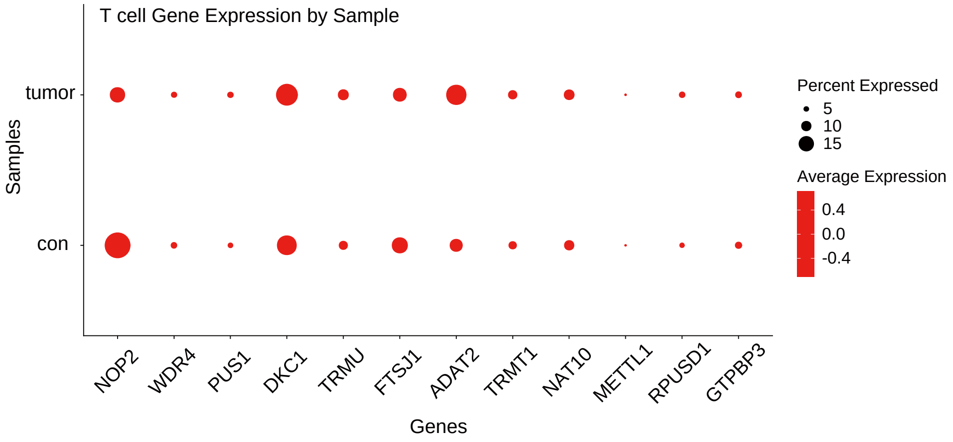

Supplementary Figure 4. Comparison of RME expression between tumor-infiltrating and normal tissue-resident T cells at the bulk population level.

Dot plot comparing the expression of 12 prognostic RME genes between tumor-infiltrating T cells and adjacent normal tissue-resident T cells without subpopulation stratification. Dot size represents the percentage of cells expressing each gene, and color intensity indicates average expression level.

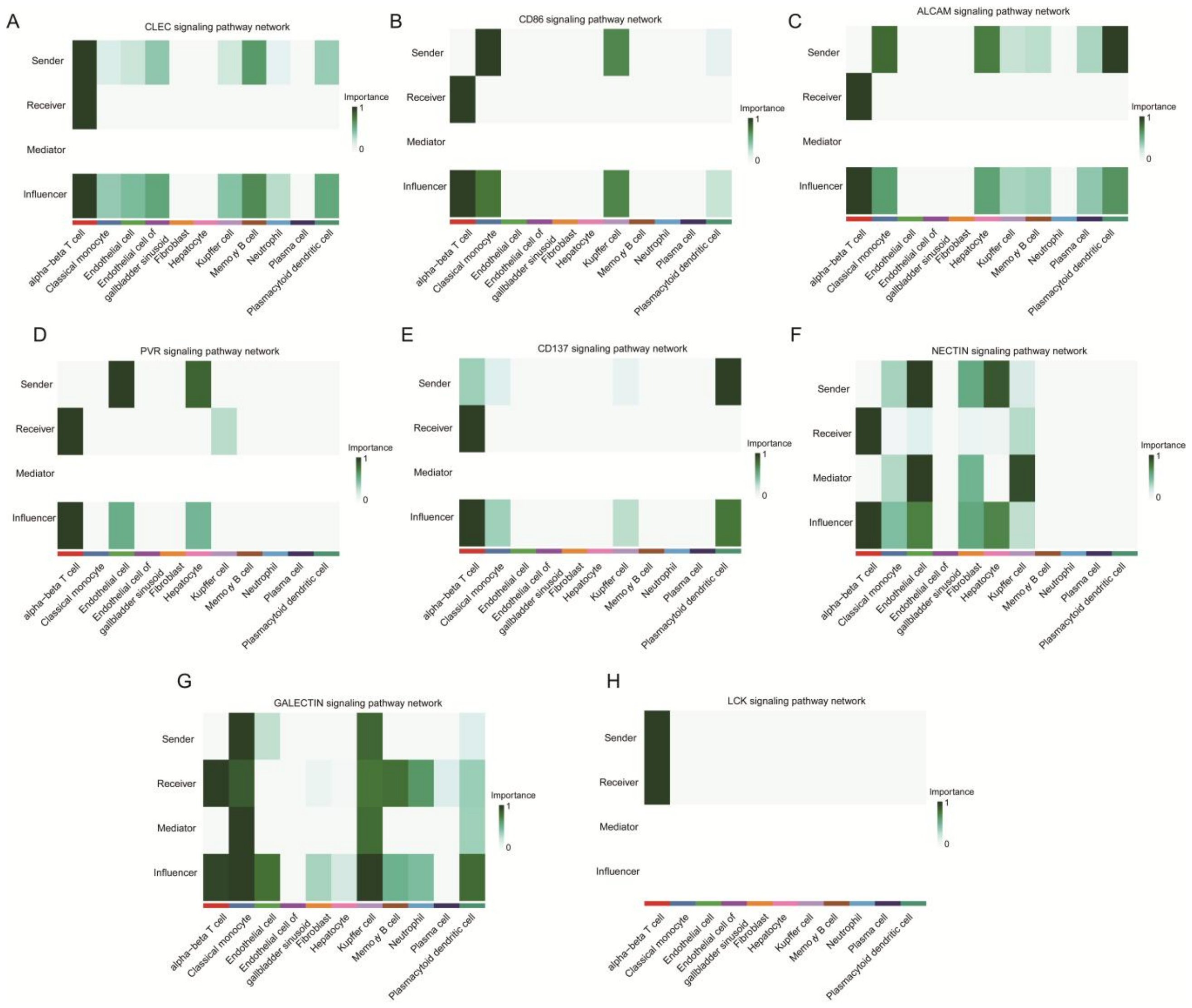

Supplementary Figure 5. Significant cell-cell communication pathways in T cell subpopulations.

Heatmaps showing eight signaling pathway networks significantly associated with T cell subpopulations: (**A**) CLEC, (**B**) CD86, (**C**) ALCAM, (**D**) PVR, (**E**) CD137, (**F**) NECTIN, (**G**) GALECTIN, and (**H**) LCK. Rows indicate cell roles (sender, receiver, mediator, influencer), and columns represent cell populations. Color intensity reflects the relative contribution to each signaling network. Alpha-beta T cells demonstrate strong communication activity across multiple pathways, underscoring their pivotal role in immune regulation within the tumor.

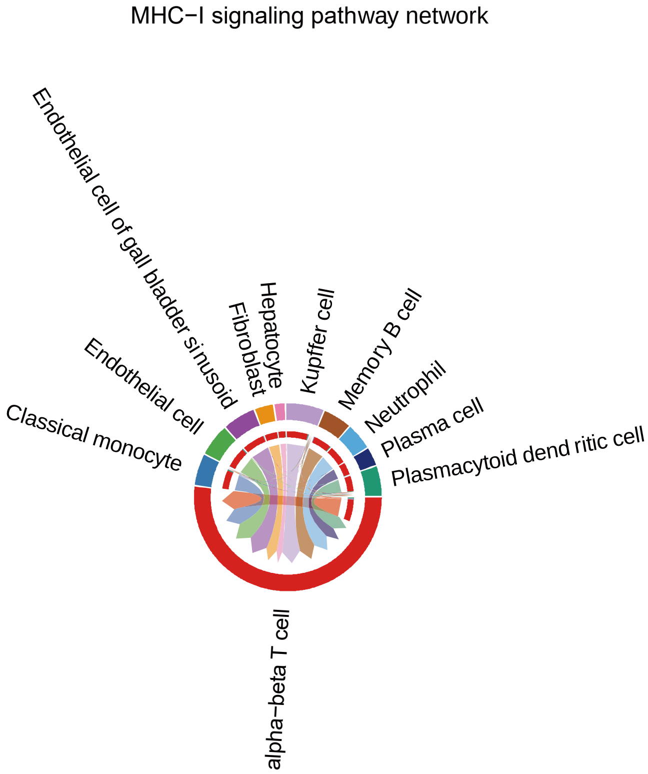

Supplementary Figure 6. MHC-I signaling pathway network inferred from cell-cell communication analysis.

Chord diagram showing predicted MHC-I-mediated interactions among cell populations. Ribbon width indicates communication strength, and outer arc length represents the contribution of each cell type as signal senders or receivers.
